# Versatile delivery platform for nucleic acids, negatively charged protein drugs, and genome-editing ribonucleoproteins using a multi-step transformable polyrotaxane

**DOI:** 10.1016/j.mtbio.2023.100690

**Published:** 2023-06-03

**Authors:** Toru Taharabaru, Takuya Kihara, Risako Onodera, Tetsuya Kogo, Yuting Wen, Jun Li, Keiichi Motoyama, Taishi Higashi

**Affiliations:** a Graduate School of Pharmaceutical Sciences, Kumamoto University, 5-1 Oe-honmachi, Chuo-ku, Kumamoto, 862-0973, Japan; b Department of Biomedical Engineering, National University of Singapore, 15 Kent Ridge Crescent, Singapore, 119276, Singapore; c Priority Organization for Innovation and Excellence, Kumamoto University, 2-39-1 Kurokami, Chuo-ku, Kumamoto, 860-8555, Japan

**Keywords:** Delivery platform, Nucleic acid and messenger RNA, Protein drug, Genome editing, Multi-step transformable polyrotaxane

## Abstract

Various biopharmaceuticals, such as nucleic acids, proteins, and genome-editing molecules, have been developed. Generally, carriers are prepared for each biopharmaceutical to deliver it intracellularly; thus, the applications of individual carriers are limited. Moreover, the development of carriers is laborious and expensive. Therefore, in the present study, versatile and universal delivery carriers were developed for various biopharmaceuticals using aminated polyrotaxane libraries. Step-by-step and logical screening revealed that aminated polyrotaxane, including the carbamate bond between the axile molecule and endcap, is suitable as a backbone polymer. Movable and flexible properties of the amino groups modified on polyrotaxane facilitated efficient complexation with various biopharmaceuticals, such as small interfering RNA, antisense oligonucleotides, messenger RNA, β-galactosidase, and genome-editing ribonucleoproteins. Diethylenetriamine and cystamine modifications of polyrotaxane provided endosomal-escape abilities and drug-release properties in the cytosol, allowing higher delivery efficacies than commercially available high-standard carriers without cytotoxicity. Thus, the resulting polyrotaxane might serve as a versatile and universal delivery platform for various biopharmaceuticals.

## Graphical abstract

**Figure:**
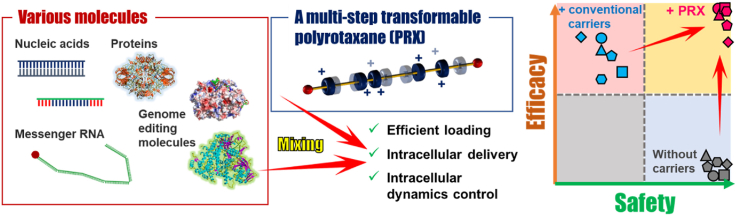

## 1 Introduction

Active pharmaceutical ingredients (APIs) have recently become more diverse, ranging from low-molecular-weight drugs to biopharmaceuticals such as protein drugs, nucleic acid drugs, and messenger RNA (mRNA) [[1], [2], [3]]. Moreover, genome editing via clustered regularly interspaced short palindromic repeats (CRISPR)-CRISPR-associated protein (Cas) has emerged as a novel therapeutic strategy for hereditary diseases [[4], [5], [6]]. Various compounds such as genes, mRNA/guide RNA, and ribonucleoproteins (RNPs) have been used for CRISPR-Cas-mediated genome editing. In most cases, these biopharmaceuticals must be internalized into the cells to exhibit their efficacy; however, the permeability of these compounds through the cell membrane is extremely low owing to their high molecular weights, hydrophilicity, and anionic surfaces. Moreover, after cellular uptake, these compounds must avoid degradation in the endo/lysosome and reach the target intracellular organelles, such as the nucleus and cytoplasm. Therefore, the development of efficient delivery systems for these compounds is urgently required, especially since the lack of efficient delivery systems is a bottleneck in the development of these biopharmaceuticals [[7], [8], [9], [10]].

To date, various carriers have been developed for the intracellular delivery of proteins and nucleic acid drugs such as small interfering RNA (siRNA), antisense oligonucleotides (ASOs), mRNA, and CRISPR-Cas compounds [[7], [8], [9], [10]]. Lipid- or polymer-based materials are often used as carrier backbones. However, in the case of lipid-based carriers, the optimization of the structure and/or composition of lipids for each biopharmaceutical should be determined. Likewise, individual structural optimization and the introduction of functional groups of polymer-based carriers are required for each biopharmaceutical [11]. Another promising strategy is to use supramolecular flameworks such as metal-organic flameworks (MOFs), covalent organic frameworks (COFs), and hydrogen-bonded organic frameworks (HOFs) as carriers for various biopharmaceuticals [[12], [13], [14]]. However, both methods of encapsulating guest drugs smaller than the host diameter of the porous material and applying a porous coating to the drug surface, require adjustment of the host diameter according to the drug or optimization of the coating process and purification method. These optimizations and functionalization are laborious but important for achieving efficient drug loading, high cellular uptake, control of intracellular dynamics, and low cytotoxicity [[15], [16], [17]]. Furthermore, the development of carriers is time-consuming, with different optimization processes needed for each molecule. Therefore, a versatile carrier applicable to a wide range of drugs and next-generation drugs is necessary to shorten the drug development process. So far, no such carrier that can be applied to various biopharmaceuticals has been reported.

Polyrotaxanes (PRXs) are mechanically interlocked supermolecules obtained by threading axile molecular chains through several cyclic molecules and subsequent endcapping of their terminals with bulky functional groups. Cyclodextrin (CD)-based PRXs have been widely reported because of their ease of preparation, high yields, wide safety profiles, low costs, and broad applications [[18], [19], [20], [21]]. For example, Ito's group has developed topological gels by cross-linking of CDs in PRXs, which exhibit various peculiar physical properties due to the “pulley effect” [22,23]. Notably, CD molecules in PRXs can rotate and move along the axile molecular chain [24]. Therefore, the functional groups modified in the CD in PRXs can move along with the CD and interact with the targeted molecules efficiently [25,26].

**Fig. 1:**
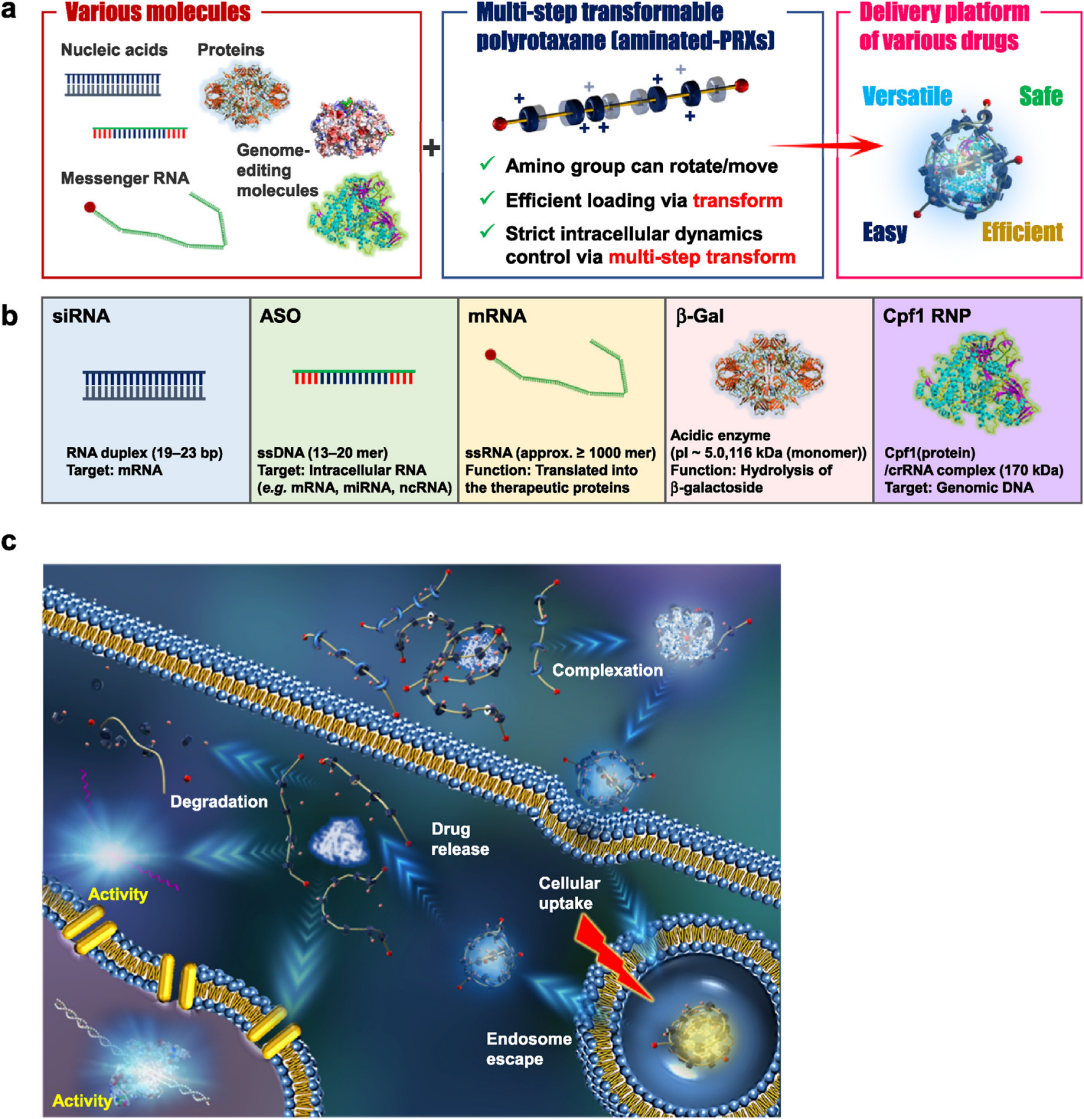
Aminated PRX-based versatile carriers for nucleic acid drugs, mRNAs, negatively charged protein drugs, and genome-editing molecules. (a) Framework of the present study; (b) properties of the biopharmaceuticals used in the present study; (c) schematic image of the multi-step transformable properties of the carriers for efficient drug delivery.

Based on these unique properties, we recently developed supramolecular pharmaceutical materials, aminated PRXs, for protein drugs and Cas9 RNP [[27], [28], [29]]. The amino groups in the CDs of aminated PRXs could interact with acidic amino acid residues of proteins or negatively charged guide RNA in Cas9 RNPs by autonomously recognizing their surface charges and molecular forms resulting from the high mobility of the amino groups. The aminated PRXs inhibited insulin adsorption onto the container and improved the physicochemical stability of the antibody [27,28]. Most importantly, the aminated PRXs could also enhance the cellular uptake of Cas9 RNP more efficiently than conventional lipid- or polymer-based carriers [29]. Therefore, the structural optimization of aminated PRX was performed to provide multi-step transformable properties in response to several intracellular environments, resulting in preferable intracellular dynamics, such as endosomal escape, release in the cytosol, and nuclear transition. The aminated PRXs achieved the highest standard in the genome editing of Cas9 RNP with negligible cytotoxicity [29]. In this structural optimization process, we assessed various structural parameters, such as the types of modified amines, biodegradable linkers, and location of the linkers, resulting in the fabrication of several aminated PRXs. This variety of parameters must work as a PRX library and be useful for the development of carriers for not only Cas9 RNP but also various biopharmaceuticals such as siRNA, ASOs, mRNA, negatively charged proteins, and other genome-editing molecules (Fig. 1a and b).

In this study, we constructed aminated PRX libraries with numerous modified amines and biodegradable linkers at different locations. The optimized aminated PRX was determined by evaluating the activity of the complexes with various compounds, such as siRNA, ASO, mRNA, β-galactosidase (β-Gal, intracellular enzyme), and Cpf1 RNP (a different type of genome-editing RNP from Cas9 RNP) (Fig. 1b). Their activities and cytotoxicities were compared with those of high-standard commercially available carriers. Moreover, we investigated the multi-step transformable properties of the drug/aminated PRX complexes to confirm the complexation and delivery mechanisms (Fig. 1c). We hypothesize that aminated PRXs can act as versatile, universal, efficient, safe, and simple delivery platforms for various biopharmaceuticals.

## 2 Materials and methods

Detailed materials and methods are provided in Supporting information. The sequences of nucleic acids are shown in Table S1.

## 3 Results and discussion

### 3.1 Construction of aminated PRX libraries

**Fig. 2:**
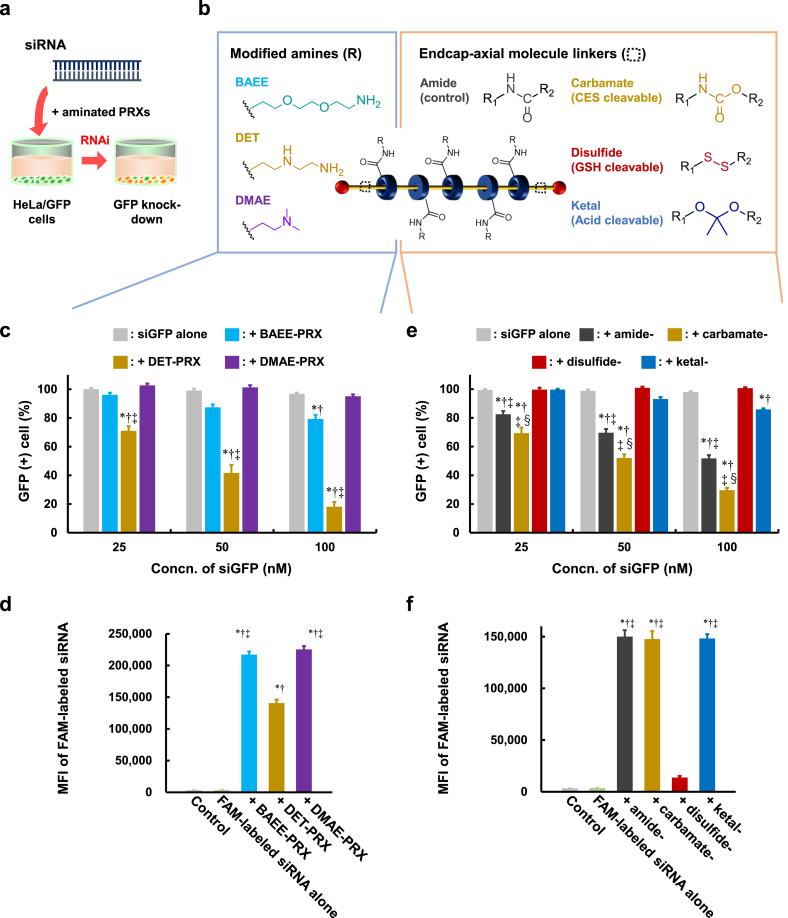
Step-by-step structural screening of aminated PRXs based on the transfection efficacies of siRNA. (a) Experimental scheme of GFP knockdown using GFP-targeted siRNA (siGFP). (b) Schematic structures of aminated PRXs with various amino groups (left) and linkers (right) between the endcap/axile molecules. (c) RNAi effects of siGFP complexes with various aminated PRXs in HeLa/GFP cells (*n* ​= ​6). ∗*p* ​< ​0.05 *vs*. siGFP alone. †*p* ​< ​0.05 *vs*. + DMAE-PRX. ‡*p* ​< ​0.05 *vs*. ​+ ​BAEE-PRX. (d) Cellular uptake of various aminated PRXs/FAM-labeled siRNA complexes in HeLa cells (*n* ​= ​5–6). [siRNA] ​= ​50 ​nM ∗*p* ​< ​0.05 *vs.* Control. †*p* ​< ​0.05 *vs.* FAM-labeled siRNA alone. ‡*p* ​< ​0.05 *vs.* ​+ ​DET-PRX. (e) RNAi effects of siGFP complexes with DET-PRXs containing various linkers between endcap/axile molecules in HeLa/GFP cells (*n* ​= ​6). ∗*p* ​< ​0.05 *vs*. siGFP alone. †*p* ​< ​0.05 *vs*. + disulfide-. ‡*p* ​< ​0.05 *vs*. + ketal-. §*p* ​< ​0.05 *vs*. ​+ ​amide-. (f) Cellular uptake of various various-DET-PRXs/FAM-labeled siRNA complexes in HeLa cells (*n* ​= ​4). [siRNA] ​= ​50 ​nM ∗*p* ​< ​0.05 *vs.* Control. †*p* ​< ​0.05 *vs.* FAM-labeled siRNA alone. ‡*p* ​< ​0.05 *vs.* ​+ ​disulfide-DET-PRX.

To develop aminated PRX libraries, various aminated PRXs were synthesized and characterized by ^1^H NMR, as reported previously [29]. Briefly, PRXs with different biodegradable linkers between the endcap and axile molecule were prepared by mixing terminal-functionalized polyethylene glycols (PEGs, M.W. 20 ​kDa) and α-CD in water, followed by endcapping with 1-adamantaneacetic acid or 1-adamantaneamine in DMF (Figs. S1–S3). In the case of ketal-containing PRXs, carboxylic acid-terminated PEG and α-CD were mixed in water and endcapped with functionalized adamantane in dimethylformamide (DMF) (Fig. S4). Next, the obtained PRXs were activated with *N,N*-carbonyldiimidazole (CDI) and reacted with 1,2-bis(2-aminoethoxy)ethane (BAEE), diethylenetriamine (DET), 2-(dimethylamino)ethylamine (DMAE), and/or cystamine (Cys) to obtain aminated PRXs (Fig. 2 and S5). The aminated PRXs prepared in this study possessed similar structural properties, such as molecular weight, the number and coverage of α-CD, and number of modified amino groups (Table S2) [29].

### 3.2 Step-by-step screening of aminated PRXs by evaluating the transfection efficacies of siRNA

**Fig. 3:**
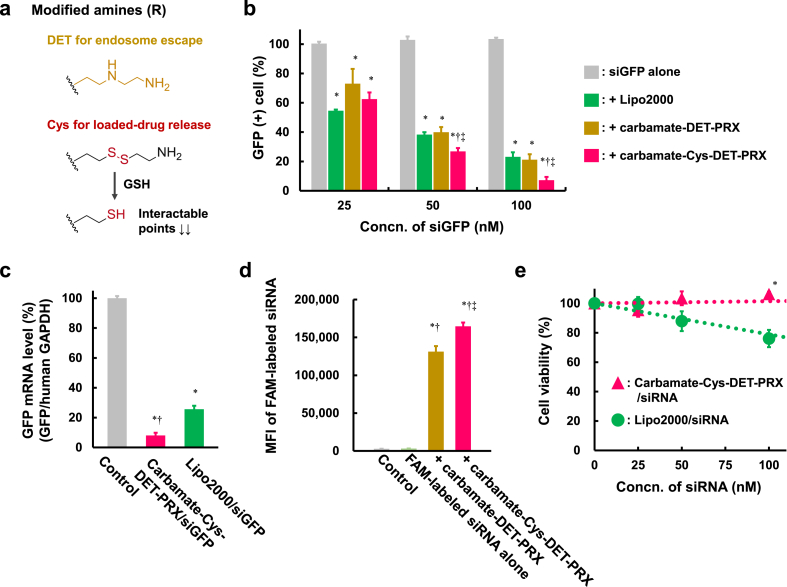
Transfection efficacy, cellular uptake, and safety of carbamate-Cys-DET-PRX/siRNA. (a) Endosomal-escaping and GSH-cleavable amines for α-CD modification. (b) RNAi effects of siGFP complexes with Lipo2000, carbamate-DET-PRX, or carbamate-Cys-DET-PRX in HeLa/GFP cells (*n* ​= ​6). ∗*p* ​< ​0.05 *vs*. siGFP alone. †*p* ​< ​0.05 *vs*. + Lipo2000. ‡*p* ​< ​0.05 *vs*. ​+ ​carbamate-DET-PRX. (c) GFP mRNA silencing of siGFP complexes in HeLa/GFP cells detected by RT-qPCR (*n* ​= ​3). [siGFP] ​= ​100 ​nM. The relative expression level of GFP/human GAPDH mRNA of non-treated control was set at 100%. ∗*p* ​< ​0.05 *vs.* control. †*p* ​< ​0.05 *vs.* Lipo2000/siGFP. (d) Cellular uptake of carbamate-DET-PRX/FAM-labeled siRNA and carbamate-Cys-DET-PRX/FAM-labeled siRNA in HeLa cells (*n* ​= ​4). [siRNA] ​= ​50 ​nM ∗*p* ​< ​0.05 *vs.* Control. †*p* ​< ​0.05 *vs.* FAM-labeled siRNA alone. ‡*p* ​< ​0.05 *vs.* ​+ ​carbamate-DET-PRX. (e) HeLa cell viability after treatment with Lipo2000/siRNA or 5G/siRNA (*n* ​= ​8). ∗*p* ​< ​0.05 *vs*. Lipo2000/siRNA.

To optimize the structure of aminated PRXs for versatile use, we initially evaluated transfection efficacies, intracellular uptake, and safety using siRNA (Fig. 2, Fig. 3). siRNA transfection efficacies were quantified by measuring green fluorescent protein (GFP) positive (+) cells after treatment with aminated PRXs/GFP-targeted siRNA (siGFP) in the human cervical epithelioid carcinoma cell line (HeLa cells) stably expressing GFP (HeLa/GFP cells) (Fig. 2a). First, we compared the siRNA transfection efficacies of aminated PRXs modified with BAEE, DET, and DMAE (Fig. 2b). BAEE is a primary amino group with a short spacer of diethylene glycol and is used as a general amino group [27,28]. DET can transform its structure from the gauche form (mono proton) to anti form (di proton) under acidic pH in late endosomes (Fig. S6), leading to late endosome-selective membrane disruption [30]. DMAE contains a tertiary amino group that can reportedly be grafted in PRX [25,26,31]. Carbamate bonds were employed as the linkers between the axile molecule and endcap of PRX in this screening step (Fig. 2b). The percentage of GFP (+) cells in HeLa/GFP cells after treatment with siGFP complexes with BAEE-, DET-, and DMAE-PRXs is shown in Fig. 2c and S7. Although siGFP alone showed a negligible effect under these experimental conditions (25–100 ​nM), the percentage of GFP (+) cells decreased by treatment with BAEE- or DET-PRXs/siGFP in siGFP concentration- (Fig. 2c) and amino units/phosphoric acid (N/P) ratio (Fig. S7)-dependent manners. In particular, the RNA interference (RNAi) effect of DET-PRX/siGFP was significantly higher than that of BAEE-PRX/siGFP and DMAE-PRX/siGFP confirmed by the percentage of GFP (+) cells (Fig. 2c) and mean fluorescence intensity (MFI) of GFP (Fig. S8). The three aminated PRXs could form complexes with siRNA under the experimental condition (data not shown) and deliver siRNA into cells (Fig. 2d). Notably, DET-PRX/siRNA had the lowest intracellular uptake among the three aminated PRXs/siRNA complexes despite having the highest RNAi effect. Hence, the highest siRNA transfection capacity of DET-PRX compared with that in other aminated PRXs is not due to differences in complex formation or cellular uptake but is a contribution of its ability to control subsequent intracellular dynamics.

As described above, DET exhibited an endosome-selective membrane disruption effect; therefore, DET-PRX likely had a high endosomal-escaping ability. In fact, the highest endosomal-escaping ability of DET-PRX/siRNA among these three types aminated PRX/siRNA complexes were confirmed by Galectin 9 (Gal9) recruitment assay which can detect the endosomal membrane disruption with high sensitivity (Fig. S9). Moreover, cell viability assay after treatment of DET-PRX at pH 7.4 or pH 5.5 suggested that membrane disruptive ability of DET-PRX is pH-dependent, and DET-PRX may selectively disrupt endosomal membranes in an acidic pH environment, but not cell membranes in a neutral pH environment (Fig. S10). Hence, we selected DET as the modified amino group in the aminated PRX. Although some PRX-based siRNA carriers have been reported thus far [26,31], DET has never been used. The results strongly support the importance of endosomal escape in siRNA delivery and provide valuable information that could facilitate the development of PRX-based siRNA carriers.

Next, the biodegradable linker between the axile molecule and endcap of PRX was optimized (Fig. 2b). Introducing a linker could trigger stimuli-responsive drug release by disrupting the structure of the PRX, leading to a decrease in the interaction points for the drugs (Fig. S11). Here, we selected four types of linkers: stable amide bonds, carboxylesterase (CES)-cleavable carbamate bonds, glutathione (GSH)-cleavable disulfide bonds, and acid-cleavable ketal bonds (Fig. 2b). The N/P ratio was set at 10 in the subsequent screening steps as the RNAi effects of all aminated PRXs/siRNAs showed the highest RNAi effect at this ratio (Fig. S7). In addition, the complexation of siRNA and the DET-PRXs were confirmed (data not shown). Both GFP (+) cell counts and MFI of GFP were decreased by treatment with amide-DET-PRX/siGFP, carbamate-DET-PRX/siGFP, and ketal-DET-PRX/siGFP in an siGFP concentration-dependent manner at N/P ratio 10 (Fig. 2e and S12). In contrast, disulfide-PRX/siGFP exhibited negligible RNAi effects, although it could be expected that the efficient siGFP release into the cytosol resulted from PRX degradation by intracellular GSH (2–10 ​mM). This was likely due to the degradation of disulfide-DET-PRX in the extracellular environment and low intracellular uptake of siRNA. In fact, amide-DET-PRX/siRNA, carbamate-DET-PRX/siRNA, and ketal-DET-PRX/siRNA showed equivalent cellular uptake. In contrast, disulfide-DET-PRX/siRNA showed significantly lower cellular uptake (Fig. 2f) because of its poor stability and release of siRNA even in the culture medium (Fig. S13). Ketal-DET-PRX also showed a lower siRNA transfection efficacy than amide-DET-PRX and carbamate-DET-PRX, even though they showed equivalent cellular uptake (Fig. 2f). Ketal-DET-PRX can degrade in the acidic endosomal environment; therefore, the polymeric structure of ketal-DET-PRX may have been disrupted in the endosome, leading to a weak endosomal-escaping ability. Most importantly, the RNAi effect of carbamate-DET-PRX/siGFP was the highest among the DET-PRXs/siGFP tested, suggesting that the carbamate bond is suitable for siRNA delivery. Carbamate bonds are enzymatically cleaved by intracellular CES [32], and carbamate-PRX can be degraded by human CES2 [29], which might contribute to not only the safety of carbamate-DET-PRX but also siRNA release. Thus, the carbamate bond was selected as the linker between the axile molecule and endcap.

To induce effective RNAi effects, siRNA must be rapidly released from the carrier into the cytosol after endosomal escape. The enzymatic degradation of carbamate-DET-PRX by CES2 is not rapid [29]; therefore, the introduction of chemically degradable linkers to aminated PRXs may enhance the release of siRNA and the effect of RNAi. Here, we introduced a chemically degradable linker—a disulfide bond—between the amino groups and CDs of aminated PRXs, as a slight degradation of the disulfide bond between the amino group and CD in the extracellular environment did not lead to the complete disruption of the PRX structure, unlike the linker between the axile molecule and endcap (Fig. S14). Therefore, we prepared Cys-modified carbamate-PRX as Cys possesses both amino groups and disulfide bonds (Fig. 3a). DET moieties were also modified to carbamate-PRX at a 1:1 ratio (Cys: DET) and termed “carbamate-Cys-DET-PRX” to assess the endosomal-escaping ability. The complex formation of carbamate-DET-PRX and carbamate-Cys-DET-PRX with siRNA were comparable (data not shown).

Then, the RNAi effects of carbamate-Cys-DET-PRX/siGFP and carbamate-DET-PRX/siGFP were evaluated (Fig. 3b, S15, and S16). As a positive control, Lipofectamine™ 2000 (Lipo2000) was employed, as it is a commercially available reagent for nucleic acid delivery. Carbamate-Cys-DET-PRX/siGFP exhibited a significantly higher RNAi effect than carbamate-DET-PRX/siGFP and Lipo2000/siGFP. The significantly more efficient RNAi effect of carbamate-Cys-DET-PRX/siGFP compared to that of Lipo2000/siGFP was confirmed more directly by quantifying GFP mRNA silencing using RT-qPCR (Fig. 3c). Although a slight enhancement of cellular uptake by the primary amine of Cys was observed (Fig. 3d), the enhancement effect of carbamate-Cys-DET-PRX/siRNA against carbamate-DET-PRX/siRNA was more modest than the RNAi effect (Fig. 3b, d, and S16). The results suggested that carbamate-Cys-DET-PRX/siRNA showed higher RNAi effect due to the improved intracellular dynamics by introducing a degradable amino group. This may have been due to the comparable stability of carbamate-Cys-DET-PRX/siRNA in the extracellular environment compared to that of carbamate-DET-PRX/siRNA (Fig. S13) and release of siRNA into the cytosol. Moreover, carbamate-Cys-DET-PRX/siRNA did not show cytotoxicity under these experimental conditions, whereas Lipo2000/siRNA did (Fig. 3e). Furthermore, non-target siRNA complexes with any carrier used in the present study did not show non-sequence-specific RNAi effects (data not shown). Hence, carbamate-Cys-DET-PRX is optimal for siRNA delivery among the various aminated PRXs in the library.

Notably, the optimized PRX (carbamate-Cys-DET-PRX) for siRNA delivery was the same as the aminated PRX (fifth generation [5G]) previously developed for Cas9 RNP delivery [29]. The structures, molecular weights, forms, and charge distributions differed between siRNA and Cas9 RNP (Fig. 1b), but carbamate-Cys-DET-PRX, namely 5G, was the optimal PRX for both siRNA and Cas9 RNP delivery. This was likely due to the transformable conformation of 5G, which allows a strong interaction between siRNA and Cas9 RNP. Moreover, the endosomal escape ability and release property of 5G are required to deliver most protein drugs and nucleic acids. Thus, 5G has potential as a versatile platform for the intracellular delivery of negatively charged protein drugs and nucleic acids.

### 3.3 Evaluation of 5G as a versatile carrier for ASOs, mRNA, negatively charged protein drugs, and genome-editing RNPs

**Fig. 4:**
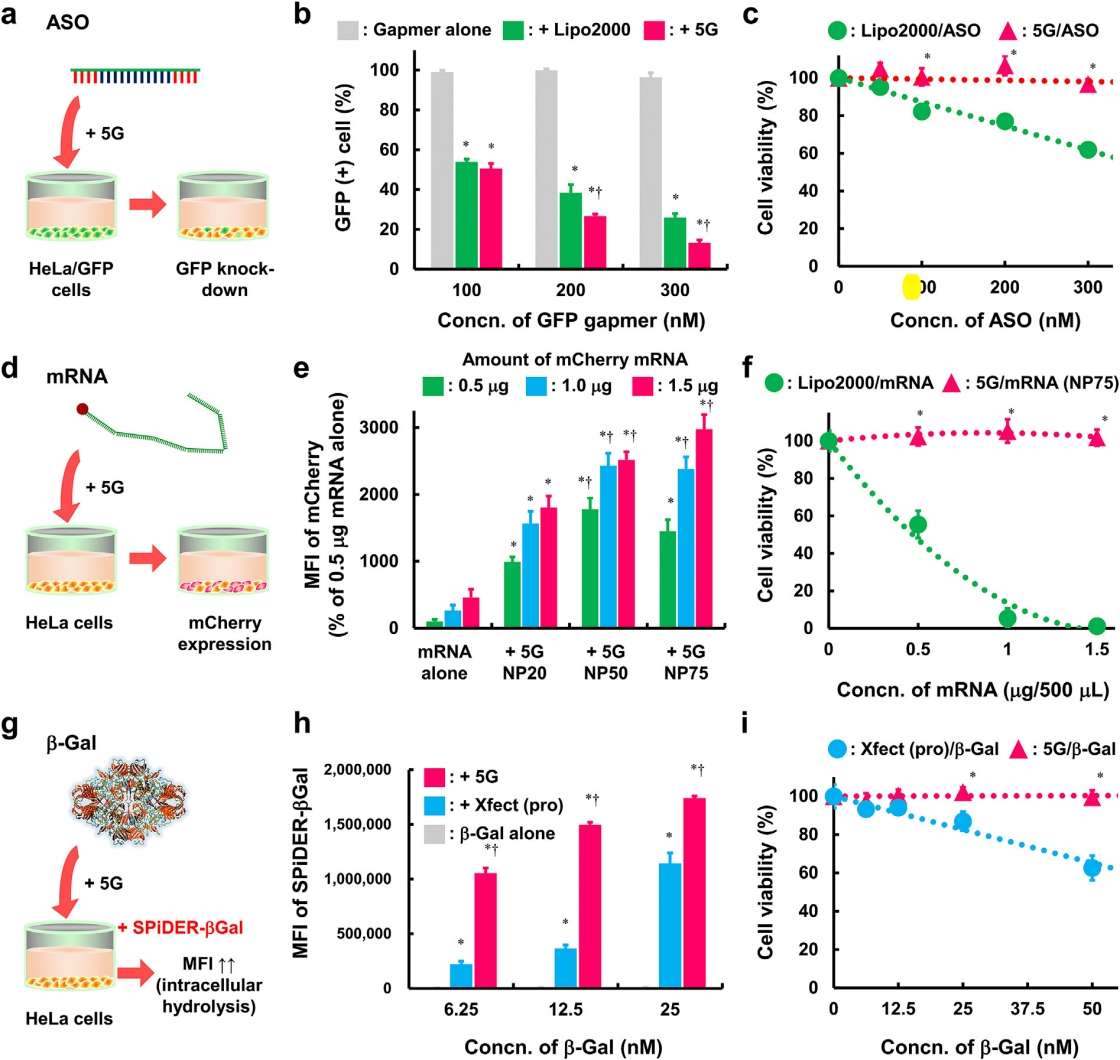
Evaluation of 5G as a carrier for ASOs, mRNA, and β-Gal based on transfection efficacy and safety. (a) Experimental scheme of GFP knockdown using GFP-targeted ASO gapmer (GFP gapmer). (b) GFP knockdown effects of GFP gapmer complexes with Lipo2000 or 5G in HeLa/GFP cells (*n* ​= ​6). ∗*p* ​< ​0.05 *vs*. GFP gapmer alone. †*p* ​< ​0.05 *vs*. ​+ ​Lipo2000. (c) HeLa cell viabilities after treatment with Lipo2000/ASO or 5G/ASO (*n* ​= ​6–8). ∗*p* ​< ​0.05 *vs*. Lipo2000/ASO. (d) Experimental scheme of mCherry mRNA transfection. (e) mCherry expression of HeLa cells after treatment with 5G/mCherry mRNA (*n* ​= ​6). ∗*p* ​< ​0.05, *vs.* mRNA alone. †*p* ​< ​0.05 *vs.* + 5G NP20. (f) HeLa cell viability after treatment with Lipo2000/mRNA or 5G/mRNA (*n* ​= ​8). ∗*p* ​< ​0.05 *vs.* Lipo2000/mRNA. (g) Experimental scheme of β-Gal delivery. (h) Intracellular enzymatic activities of β-Gal complexes with Xfect (pro) or 5G in HeLa/GFP cells (*n* ​= ​4). ∗*p* ​< ​0.05 *vs*. β-Gal alone. †*p* ​< ​0.05 *vs*. ​+ ​Xfect (pro). (i) HeLa cell viability after treatment with Xfect (pro)/β-Gal or 5G/β-Gal (*n* ​= ​8). ∗*p* ​< ​0.05 *vs*. Xfect (pro)/β-Gal.

**Fig. 5:**
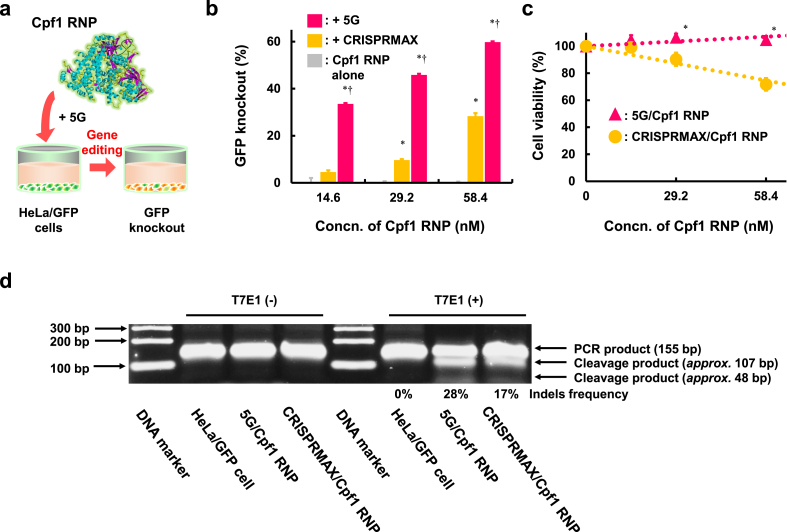
Evaluation of 5G as a carrier for Cpf1 RNP based on transfection efficacy and safety. (a) Experimental scheme of GFP knockout using GFP-targeted Cpf1 RNP. (b) Genome-editing activity of Cpf1 RNP complexes with CRISPRMAX or 5G in HeLa/GFP cells (*n* ​= ​3). ∗*p* ​< ​0.05 *vs*. Cpf1 RNP alone. †*p* ​< ​0.05 *vs*. ​+ ​CRISPRMAX. (c) HeLa cell viability after treatment with CRISPRMAX/Cpf1 RNP or 5G/Cpf1 RNP (*n* ​= ​8). ∗*p* ​< ​0.05 *vs*. CRISPRMAX/Cpf1 RNP. (d) T7E1 assay of the target gene (GFP) after treatment of 5G/Cpf1 RNP or 5G/CRISPRMAX. The indels frequencies were quantified using ImageJ software.

To evaluate the potential of 5G, carbamate-Cys-DET-PRX, as a versatile and universal platform for the intracellular delivery of negatively charged protein drugs and nucleic acids, their ability to deliver ASOs, mRNA, negatively charged protein (β-Gal), and other genome-editing RNP (Cpf1 RNP) and safety of the biopharmaceutical-loaded 5G complexes was evaluated (Fig. 4, Fig. 5).

ASOs can suppress the function of intracellular RNA, such as mRNA, microRNAs (miRNAs), and non-coding RNA (ncRNAs). ASOs can be slightly permeable through the cell membrane in contrast to other nucleic acids such as siRNA. However, high concentrations and long processing times are required for their intracellular uptake; therefore, the efficiency of ASOs is enhanced using carriers. We prepared 5G complexes with GFP-targeted ASO (GFP ASO) and GFP-targeted gapmer type ASO (GFP gapmer) and evaluated their mRNA knockdown efficacies by measuring GFP (+) cells and MFI of GFP in HeLa/GFP (Fig. 4a). Under the present experimental conditions, GFP ASO and GFP gapmer alone did not exhibit GFP knockdown (Fig. 4b, S17–19). Both 5G/GFP ASO and 5G/GFP gapmer showed GFP knockdown in an ASO/gapmer concentration- and N/P ratio-dependent manner (Fig. 4b, S17–19) without non-sequence-specific effect (data not shown). In addition, 5G/GFP ASO and 5G/GFP gapmer induced significantly stronger GFP knockdown than Lipo2000/GFP ASO and Lipo2000/GFP gapmer, respectively (Fig. 4b, S17, S18, S20). The significantly more efficient gene silencing effect of 5G/GFP gapmer compared to that of Lipo2000/GFP gapmer was confirmed more directly by quantifying GFP mRNA levels using RT-qPCR (Fig. S21). 5G/ASO did not show cytotoxicity, whereas Lipo2000/ASO did (Fig. 4c). These results demonstrate the potential of 5G as a high-standard carrier for ASOs.

mRNA is a powerful tool for introducing various therapeutic proteins into cells. Recently, mRNA-based vaccines have attracted considerable attention for preventing coronavirus disease 2019 [33]. Therefore, we evaluated the potential of 5G as an mRNA carrier using mCherry mRNA (Fig. 4d). The fluorescence derived from mCherry in HeLa cells was significantly enhanced by complexation with 5G in N/P ratio and mRNA concentration-dependent manners (Fig. 4e). Lipo2000/mRNA showed marked cytotoxicity, even with 0.5 ​μg of mRNA. In contrast, 5G/mRNA did not show cytotoxicity, even with 1.5 ​μg of mRNA (Fig. 4f). Thus, 5G is useful as an mRNA carrier.

Approximately 20% of the drugs on the market are proteins, such as monoclonal antibodies, cytokines, growth factors, and insulin [34]. However, many commercially available protein drugs have been developed for extracellular targets owing to the poor membrane permeability of proteins. Meanwhile, more than 60% of the pathways that control cellular functions involve intracellular events [35]. Thus, the intracellular delivery of protein drugs can expand their functions. Hence, we evaluated the potential of 5G as an intracellular protein carrier. β-Gal, an anionic enzyme, was selected as a model protein as it is a candidate compound for enzymatic prodrugs [36] and enzyme replacement therapies [37,38]. To evaluate the delivery efficacy of 5G, the fluorescence of SPiDER-βGal in HeLa cells was measured after treatment with 5G/β-Gal (Fig. 4g). SPiDER-βGal is a reagent that can only be detected by the intracellular enzymatic activity of β-Gal. The intracellular enzymatic activity of 5G/β-Gal was significantly higher than that of β-Gal alone and of the complex with the Xfect™ Protein Transfection Reagent (Xfect [pro]), a commercially available positive control (Fig. 4h, S22, and S23). Moreover, the cell viability assay suggested that 5G/β-Gal was safer than Xfect (pro)/β-Gal (Fig. 4i). These results indicate the potential of 5G as an intracellular delivery carrier for negatively charged proteins. In this study, we evaluated the delivery ability of 5G for β-Gal (isoelectric point: ∼5.0), but the relationship between the isoelectric point, *i.e.,* charge state in neutral buffer of the loaded protein drugs and its delivery ability by 5G needs to be investigated. The movable property of 5G may favor interaction with surface anionic amino acid residues even if the total charge of the protein is positive, which may be more tolerant of surface charge differences than conventional cationic polymers.

Recently, various CRISPR tools have been developed as alternatives to Cas9-based methods [39]. For example, the Cpf1 enzyme can act on genomic regions in which Cas9 cannot as the Cpf1 system proficiently cleaves target DNA adjacent to a short T-rich PAM (TTTV or TTTN), whereas Cas9 acts on a G-rich PAM (NGG). Although both Cas9 and Cpf1 are preferentially delivered in cells as RNPs, it is unclear whether 5G can deliver Cpf1 RNPs. In fact, Cpf1 RNPs possess few anionic functional groups and a weak negative charge when compared with Cas9 RNPs. Therefore, we evaluated the potential of 5G as a Cpf1 RNP carrier. Lipofectamine™ CRISPRMAX™ (CRISPRMAX), a commercially available high-standard reagent for Cas9 RNP transfection, was used as a positive control. Genome-editing efficacies were quantified by measuring the GFP knockout frequency and MFI of GFP in HeLa/GFP cells (Fig. 5a). As a result, 5G/Cpf1 RNP showed genome editing in Cpf1 RNP concentration- and amino units/(phosphoric acid of crRNA ​+ ​carboxylic acid of Cpf1 protein) (N/[P ​+ ​C]) ratio-dependent manners (Fig. 5b, S24, and S25). In addition, 5G/Cpf1 RNP induced highly efficient genome editing (Fig. 5b, S25, and S26) with high safety (Fig. 5c) compared with CRISPRMAX/Cpf1 RNP. The genome editing effects of 5G/Cpf1 RNP and CRISPRMAX/Cpf1 RNP were confirmed by T7E1 assay, and suggesting that 5G/Cpf1 RNP also induced highly efficient genome editing compared with CRISPRMAX/Cpf1 RNP in target DNA mutation level (28% and 17%, respectively) (Fig. 5d). The significantly high inhibitory effect of 5G/Cpf1 RNP on GFP mRNA production compared to CRISPRMAX/Cpf1 RNP was confirmed by quantifying GFP mRNA levels using RT-qPCR (Fig. S27). These results highlight the potential of 5G as a Cpf1 RNP carrier. CRISPRMAX was tuned for Cas9 RNP, and the structure of Cpf1 RNP differs from that of Cas9 RNP. This likely resulted in the low delivery efficacy of Cpf1 RNP. By contrast, 5G possesses autonomous transforming properties that allow efficient complexation with Cpf1 RNP, resulting in high genome-editing efficiency. As future efforts, we should study the therapeutic potential of 5G/genome-editing RNP system for the treatment of genetic diseases which can be cured by single-gene knockout, such as sickle cell disease [40], transthyretin amyloidosis [41], and familial hypercholesterolemia [42]. Moreover, next-generation genome-editing tools, such as CRISPR activation [43], base editors [44], and prime editors [45], have been developed recently. To achieve next-generation genome editing, various types of genome-editing molecules should be delivered in the cell, which may be accomplished with the autonomous transforming properties of 5G. In conclusion, 5G could be useful as a high-standard, safe, and versatile delivery carrier for nucleic acid drugs, mRNA, negatively charged proteins, and genome-editing molecules.

### 3.4 Multi-step transformable properties of 5G for various drugs

**Fig. 6:**
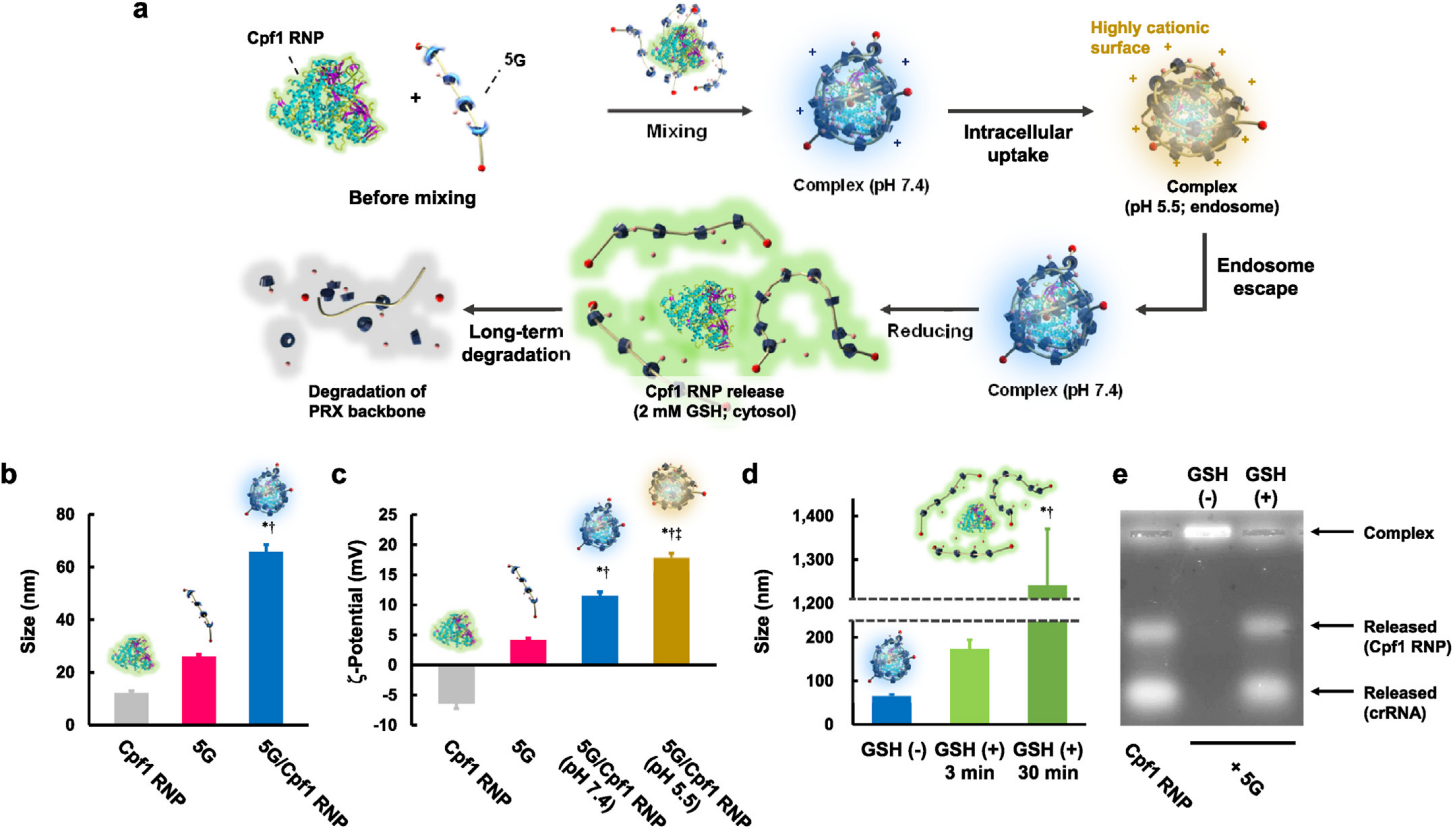
Multi-step transformable properties of 5G complexes with Cpf1 RNP. (a) Schematic representation of the multi-step transformable properties of 5G in various extra/intracellular environments. (b) Sizes and (c) ξ-potentials of Cpf1 RNP, 5G, and 5G/Cpf1 RNP in HBSS (pH 7.4) or acetate buffer (pH 5.5) (*n* ​= ​4). ∗*p* ​< ​0.05 *vs*. Cpf1 RNP. †*p* ​< ​0.05 *vs*. 5G. ‡*p* ​< ​0.05 *vs*. 5G/Cpf1 RNP (pH 7.4). (d) Effect of GSH on 5G/Cpf1 RNP size (*n* ​= ​4). [GSH] ​= ​2 ​mM ∗*p* ​< ​0.05 *vs*. GSH (−). †*p* ​< ​0.05 *vs*. GSH (+), 3 ​min. (e) Agarose gel electrophoresis of 5G/Cpf1 RNP with or without GSH (2 ​mM) treatment for 30 ​min.

The superior delivery efficacies for various biopharmaceuticals and high safety of 5G are likely because of its multi-step transformable properties, namely 1) its efficient complexation ability through the moving properties of amino groups, 2) the diprotonation of DET and strong membrane disruption ability in the endosomes, 3) degradation of Cys for drug release in the cytosol, and 4) long-term degradation of the PRX backbone (Fig. 1, Fig. 6a). The physicochemical properties of 5G complexes with siRNA, ASOs, mRNA, β-Gal, and Cpf1 RNP were examined under various stimulus environments to verify the transformable properties. Complex formation of 5G with all biopharmaceuticals used in the present study (siRNA, ASO, mRNA, β-Gal, and Cpf1 RNP) was confirmed (Fig. 6, S28–S31), suggesting that 5G can load a wide range of biopharmaceuticals due to its autonomous transforming properties. Representative data using Cpf1 RNP are shown in Fig. 6b–e. Cpf1 RNP and 5G autonomously formed complexes with a diameter of approximately 65 ​nm (Figs. 6b) and 12 ​mV of ζ-potential (Fig. 6c) by mixing only. The ζ-potential of the 5G/Cpf1 RNP at a pH of 5.5, which resembled a typical endosomal environment, was higher than that at pH 7.4. This was likely due to the diprotonation of DET (Fig. 6c). Furthermore, the particles were disrupted in the presence of 2 ​mM GSH, which is a typical intracellular concentration, likely due to degradation of Cys moieties (Fig. 6d). The larger particle size of 5G/Cpf1RNP in response to GSH was likely due to the very weak interaction between reduced Cys-DET-PRX and Cpf1 RNP resulting in the aggregation in the absence of anionic competitor unlike the cellular circumstances, and/or thiol-thiol cross-linking of reduced Cys-DET-PRX. In fact, the agarose gel electrophoresis of 5G/Cpf1 RNP in the presence both of GSH and heparin, an electrostatic competitor, suggested the effective release in the response to GSH, while the complex was stable in the presence of heparin alone (Fig. 6e). These transformable properties were also confirmed in 5G/siRNA, 5G/ASO, 5G/mRNA, and 5G/β-Gal (Figs. S28–32). These results indicate the multi-step transformable properties of 5G complexes in cells.

Overall, 5G could form complexes efficiently and load various biopharmaceuticals due to its autonomous transforming properties. Previous studies have demonstrated that CDs in PRXs are movable along threading PEG [24]. Moreover, most recently, we have demonstrated that aminated-PRXs can rapidly neutralized the negative charge of insulin or Cas9 RNP, *i.e.*, can form complexes much more easily than the unmovable control polymer with comparable molecular weights and amino group modification rates to the aminated PRX [27,29]. Furthermore, the molecular level interaction of the aminated PRX and Cas9 RNP was confirmed by cryo-transmission electron microscopy (cryo-TEM) observation [29]. The broad applicability of 5G revealed in this study is based on the complex formation due to these dynamic properties. As future efforts, the ultrastructure and elemental distribution of 5G complexes with various biopharmaceuticals should be studied in more detail by using TEMs.

Thus far, various promising non-viral delivery systems have been developed by using lipid-based, polymer-based, and/or supramolecular flameworks-based materials. These conventional carrier systems require optimization of the carrier itself, mixing ratios of multiple carrier components, and preparation/purification protocols for each biopharmaceutical [[7], [8], [9], [10], [11], [12], [13], [14], [15], [16], [17]]. The most important difference between 5G system and such carrier systems is its simplicity: 5G system can be easily prepared by simply mixing the loaded drug and a single 5G compound. Therefore, the mixing ratio can be easily optimized in the early stages of the study, as shown in Figs. S7, S19, S22, and S24.

In addition to complex formation, 5G can control the intracellular dynamics of loaded drugs through multi-step transformable properties in response to the intracellular environment, thereby ensuring the pharmacological activity of loaded drugs in the cytosol. Including our previous reports, at least, 5G is useful in the delivery of 6 anionic molecules with completely different structures and molecular weights: Cas9 RNP [29], siRNA, ASO, mRNA, β-Gal, and Cpf1 RNP (Table S3). Furthermore, similar aminated PRXs (BAEE-PRX in Fig. 2 [28] and PEGylated BAEE-PRX [27]) have also proved its usefulness in the formulation of insulin and IgG, and the scope of 5G application could be even broader. The ability to deliver a wide range of biopharmaceuticals as a single agent is one of the advantages of 5G (Table S3).

All 5G complexes with various biopharmaceuticals were confirmed their higher safety compared to commercially available reagents such as Lipo2000, Xfect (pro), and CRISPRMAX complexes *in vitro* within the treatment concentrations at which they were pharmacologically effective (Fig. 3, Fig. 4, Fig. 5c)*.* Despite its potential cationic-mediated cytotoxicity, the safeties of 5G complexes were likely due to the high safety of modified DET at pH 7.4 as shown in Fig. S10. In fact, the complexes of DET-modified polymers and plasmid DNA were reported that they do not show cytotoxicity even at high N/P ratios [46]. Furthermore, we have recently revealed that aminated PRXs with PEG as the axile molecule, such as 5G, form hydrated layer on the surface of complexes with proteins/nucleic acids (unpublished but submitted data). In fact, copolymer of DET-modified polymers with PEG makes the cytotoxicity of the DET-modified polymer/plasmid DNA complex even more permissive [46], so such PEG hydrated layer formation may contribute to the high safety of 5G complexes. However, we should note that our safety studies were limited to *in vitro*. We need to examine the multiple *in vivo* injections of each 5G complex for more careful safety studies and investigate the production of 5G-complexes-specific antibodies by activation of T cells, PEG-specific IgG and IgM [47], as well as complement activation-related pseudoallergy response (CARPA) [48,49]. Particular attention should be paid to complement activation, as it has been reported to be strongly dependent on cationic polymer chain length [50].

## 4 Conclusion

In this study, we developed high-standard, safe, and versatile delivery carriers for nucleic acid drugs, mRNA, negatively charged proteins, and genome-editing molecules using aminated PRX libraries. Carbamate-Cys-DET-PRX, namely aminated PRX (5G), universally acted as an efficient carrier for siRNA, ASOs, mRNA, β-Gal, and Cpf1 RNP, despite the wide variety of structures, molecular weights, forms, and charge distributions of these compounds, which were likely due to the multi-step transformable properties of 5G. *In vivo* human studies and careful safety evaluations are essential for future efforts. Recently, various biopharmaceuticals have been actively developed. However, the preparation of intracellular carriers for each biopharmaceutical is laborious. It is very meaningful to develop effective carriers for various biopharmaceuticals, and utility of 5G as a universal carrier was demonstrated in this study. Moreover, 5G-based strategies may facilitate and shorten the formulation process for such biopharmaceuticals and meet the need for rapid drug development, such for novel-developed technology or during pandemics. Our results indicate that 5G can be a versatile and universal delivery platform for various biopharmaceuticals.

## Statement of significance

In this study, we developed a carrier that can be freely transformed to match the shape and charge distribution of various biopharmaceuticals, such as siRNA, ASO, mRNA, anionic protein, and Cas12a (Cpf1) RNP. This carrier showed better safety and transfection efficiency than the high-standard existing commercial products. Until now, it has been common practice to optimize carriers for each drug, but with our findings, we have developed an all-in-one type carrier for various biopharmaceuticals. To the best of our knowledge, this is the first such carrier, and we believe that its novelty and scientific impact will be extremely high.

## Author contributions

Toru Taharabaru: Conceptualization, funding acquisition, data curation, formal analysis, investigation, methodology, validation, writing – original draft, and writing – review and editing. Takuya Kihara: Investigation, methodology, and validation. Risako Onodera, Tetsuya Kogo, and Yuting Wen: Methodology and validation. Jun Li and Keiichi Motoyama: Methodology and supervision. Taishi Higashi: Conceptualization, funding acquisition, investigation, methodology, supervision, writing – original draft, and writing – review and editing. All authors have given approval to the final version of the manuscript.

## Declaration of competing interest

The authors declare that they have no known competing financial interests or personal relationships that could have appeared to influence the work reported in this paper.

## Data Availability

Data will be made available on request.
