# Supplementary material for: Versatile delivery platform for nucleic acids, negatively charged protein drugs, and genome-editing ribonucleoproteins using a multi-step transformable polyrotaxane
Source: Mater Today Bio. 2023 Jun 3;20:100690. doi: 10.1016/j.mtbio.2023.100690 (PMC10333717; doi:10.1016/j.mtbio.2023.100690)
Supplement: Multimedia component 1 [file mmc1.pdf]

Supporting Information

Versatile delivery platform for nucleic acids, negatively charged protein drugs, and genome-editing ribonucleoproteins using a multi-step transformable polyrotaxane

Toru Taharabaru[a, b], Takuya Kihara[a], Risako Onodera[a], Tetsuya Kogo[a], Yuting Wen[b], Jun Li[b], Keiichi Motoyama[a], and Taishi Higashi[a, c*]

[a]Graduate School of Pharmaceutical Sciences, Kumamoto University, 5-1 Oe-honmachi, Chuo-ku, Kumamoto 862-0973, Japan

[b]Department of Biomedical Engineering, National University of Singapore, 15 Kent Ridge Crescent, Singapore 119276, Singapore

[c]Priority Organization for Innovation and Excellence, Kumamoto University, 2-39-1 Kurokami, Chuo-ku, Kumamoto 860-8555, Japan

* Corresponding author.

Taishi Higashi

Email: higashit@kumamoto-u.ac.jp

TEL: +81 96 371 4168, FAX; +81 96 371 4168

Materials and Methods

Materials

α-CD was supplied by Nihon Shokuhin Kako (Tokyo, Japan). Cells from the human cervical epithelioid carcinoma cell line HeLa were obtained from Riken Bioresource Center (Tsukuba, Japan). HeLa cells stably expressing green fluorescent protein (GFP; HeLa/GFP) were obtained from Cell Biolabs, Inc. (San Diego, CA, USA). Small interfering RNA (siRNA) was ordered from Hokkaido System Science Co., Ltd. (Sapporo, Japan). Antisence oligonucleotides (ASOs), Cpf1 protein, and CRISPR RNA for Cpf1 (crRNA) were purchased from Integrated DNA Technologies Japan (Tokyo, Japan). The sequences of the siRNA, ASOs, and crRNA targets are shown in Table S1. mCherry mRNA was obtained from Funakoshi Co., Ltd. (Tokyo, Japan). Opti-MEM, Lipofectamine™ 2000 (Lipo2000), Lipofectamine™ CRISPRMAX™ (CRISPRMAX), BLOCK-iT™ Fluorescent Oligo, and β-galactosidase (β-Gal) were obtained from Thermo Fisher Scientific K.K. (Tokyo, Japan). Xfect™ Protein Transfection Reagent (Xfect [pro]) was purchased from Takara Bio (Shiga, Japan). SPiDER-βGal and Cell Counting Kit-8 were obtained from Dojindo (Kumamoto, Japan).

Preparation of various aminated polyrotaxanes (PRXs)

The aminated PRXs were prepared and characterized by ^{1}H-NMR as previously described [1]. Briefly, the terminals of polyethylene glycol (PEG; 20 kDa; Sigma-Aldrich, Tokyo, Japan) were functionalized with various biodegradable moieties in a round-bottom flask with rubber cap and stir by a stirrer and a magnetic stirrer bar. In a beaker with stir by a stirrer and a magnetic stirrer bar, functionalized PEG and α-CD were mixed in water to obtain polypseudorotaxanes (PPRXs). The PPRXs gel were transferred into a centrifuge tube using a medicine spoon, centrifuged, and

the precipitate was collected and lyophilized. In an Erlenmeyer flask with rubber cap and stir by a stirrer and a magnetic stirrer bar, lyophilized PPRXs were capped with 1-adamantaneacetic acid or 1-adamantaneamine in dimethylformamide (DMF) to obtain PRXs with various biodegradable bonds between the axial molecules and endcaps. The hydroxyl groups of α-CD in the PRXs were activated with *N,N*-carbonyldiimidazole (CDI) in an Erlenmeyer flask with rubber cap and stir by a stirrer and a magnetic stirrer bar. Amino groups such as 1,2-bis(2-aminoethoxy)ethane (BAEE), diethylenetriamine (DET), 2-(dimethylamino)ethylamine (DMAE), and/or cystamine (Cys) dihydrochloride were made to react with the activated PRXs in an Erlenmeyer flask with rubber cap and stir by a stirrer and a magnetic stirrer bar. In the case of ketal-PRX, α-CD could not be threaded onto the ketal-terminated PEG. Therefore, 1-adamantaneacetic acid was reacted with 2,2-bis(aminoethoxy)propane to obtain 1-adamantane ketal in a round-bottom flask with rubber cap and stir by a stirrer and a magnetic stirrer bar. Carboxylic acid-terminated PPRX was then capped with the 1-adamantane ketal. The detailed synthesis schemes and the characterization results reported previously [1] are shown in Figure S1–S5 and Table S2, respectively.

Cell culture

HeLa and HeLa/GFP cells were cultured in high-glucose Dulbecco’s modified Eagle’s medium (DMEM) containing 2 mM of glutamine, 100 U/mL of penicillin, 100 mg/L of streptomycin, and 10% fetal bovine serum (FBS). Then, 10 μg/mL of blasticidin was added to the HeLa/GFP cells. The cells were maintained at 37 °C in a 5% CO2 atmosphere in the cell culture incubator. All cell culture experiments were conducted in a biosafety cabinet.

Evaluation of transfection efficacy

HeLa/GFP cells (3.75 × 10^4 cells/well in a 24-well plate) were seeded 24 h before transfection. The cells were then washed twice with serum-free medium. In a 1.5 mL Eppendorf tube, aminated PRXs were mixed and incubated for 15 min with siRNA, ASO, β-Gal, or Cpf1/crRNA (Cpf1 RNP) in Opti-MeM. The detailed charge ratios, namely, amino units/phosphoric acid (N/P), amino units/carboxylic acid (N/C), and amino units/(phosphoric acid + carboxylic acid) (N/[P+C]), and detailed transfection protocols for each molecule are described below.

***siRNA and ASO complexes*:** The N/P ratio of aminated PRX and siRNA or ASO was set to 10. The volume-to-amount ratio of Lipo2000 to siRNA or ASO was set to 3.75. Three hundred microliters of siRNA or ASO complexes in DMEM was added to each well by using a P1000 pipette. The samples were then incubated at 37 °C for 4 h and washed twice with DMEM. The cells were cultured for 68 h in DMEM (500 μL; 10% FBS). After washing twice with HBSS, the cells were scraped into HBSS. After centrifugation, the cells were dispersed in 1 mL of 10% FBS/HBSS. After filtration with nylon mesh, the cells (1 × 10^4 cells) were analyzed using a BD Accuri C6 flow cytometer (BD Biosciences Japan, Tokyo, Japan) and BD Accuri C6 software (BD Biosciences). The fluorescence images were obtained by using a fluorescence microscope (Biorevo BZ-9000, Keyence, Osaka, Japan).

mRNA Complexes: The N/P ratio of aminated PRX and mRNA was set to 20–75. The volume-to-amount ratio of Lipo2000 to mRNA was set to 3.75. The mRNA complexes in DMEM (500 μL) were added to each well by using a P1000 pipette. After incubation at 37 °C for 4 h, the cells were washed twice with DMEM. Fresh DMEM (500 μL; 10% FBS) was then added. The cells were cultured for 44 h and analyzed using a flow cytometer. The mean fluorescence intensity (MFI) of mCherry was represented as the percentage of that of 0.5 μg of mRNA alone.

***β-Gal complexes*:** The N/C ratio of aminated PRX and β-Gal was set to 5. Xfect (pro)/β-Gal was prepared according to the manufacturer's protocol. β-Gal complexes in DMEM (500 μL) were then added to each well and incubated at 37 °C for 4 h by using a P1000 pipette. The concentration of β-Gal was represented as that of the monomer. After washing twice with HBSS, the cells were scraped into HBSS. After centrifugation, the cells were dispersed in SPiDER-βGal working solution (1:1000 diluted SPiDER-βGal stock solution in HBSS) in a 1.5 mL Eppendorf tube and incubated at 37 °C for 15 min on a block incubator. After centrifugation, the cells were dispersed in fresh HBSS. This process was repeated twice. The cells were then analyzed with a flow cytometer, and the MFI of SPiDER-βGal was calculated by subtracting the MFI of β-Gal-untreated cells with SPiDER-βGal from each sample. The fluorescence images were obtained by using a fluorescence microscope (Biorevo BZ-9000, Keyence, Osaka, Japan).

***Cpf1 RNP complexes*:** The N/(P+C) ratio of aminated PRX to Cpf1 RNP was set to 10. CRISPRMAX/Cpf1 RNPs were prepared according to the manufacturer protocol for Cas9 RNPs. Cpf1 RNP complexes in DMEM (500 μL) were added to each well and incubated at 37 °C for 4 h by using a P1000 pipette. After washing with DMEM twice and adding fresh DMEM (500 μL; 10% FBS), the cells were cultured for 5 days and analyzed using a flow cytometer. GFP knockout (%) was calculated using the following equation: GFP knockout (%) = 100 − 100 × (GFP positive cells in the sample/that of untreated control). The fluorescence images were obtained by using a fluorescence microscope (Biorevo BZ-9000, Keyence, Osaka, Japan).

Intracellular uptake of siRNA complexes

HeLa cells (3.75×10^4 cells/well in a 24-well plate) were seeded 24 h before transfection. The cells were then washed twice with serum-free medium. Aminated PRXs were mixed and incubated for

15 min with the BLOCK-iT^{TM} Fluorescent Oligo (fluorescein [FAM]-labeled siRNA) in a 1.5 mL Eppendorf tube. The N/P ratio of aminated PRX and siRNA was set to 10. Three hundred microliters of siRNA complexes in DMEM were added to each well by using a P1000 pipette. The samples were then incubated at 37 °C for 4 h and washed twice with HBSS. Subsequently, the cells were washed with HBSS twice, trypsinized, collected by centrifugation, dispersed in 500 µL of 10% FBS containing HBSS, and filtered through a nylon mesh. Ten thousand cells were analyzed using a BD Accuri C6 flow cytometer (BD Biosciences Japan, Tokyo, Japan) and BD Accuri C6 software (BD Biosciences).

Galectin 9 (Gal9) recruitment assay

HeLa cells (1.5×10^4 cells/well in the center glass of a 35 mm glass base dish) were seeded 24 h before transfection. After the cells were washed twice with serum-free medium, siRNA complexes in DMEM (200 µL) were added to each well and incubated at 37 °C for 4 h. The Gal9 staining was conducted as per previous report [1]. The stained Gal9 foci were observed using a confocal laser scanning microscope (Leica TCS SP5, Leica Japan, Tokyo, Japan). The number of Gal-9 foci/cells in the images was then quantified.

Cytotoxicity

HeLa cells (1.5×10^4 cells/well in a 96-well plate) were seeded 24 h before transfection. The cells were then washed twice with serum-free medium. Carbamate-Cys-DET-PRX complexes in DMEM (200 µL, at the same concentration as in the transfection experiments) were added to each dish by using a P200 pipette. After incubation at 37 °C for 4 h and washing twice, fresh DMEM (200 µL; 10% FBS) was added to each well. After incubation at 37 °C for 20 h, the cells were

washed twice with HBSS. HBSS (100 μL) and Cell Counting Kit-8 reagent (10 μL; Dojindo, Kumamoto, Japan) were added to each well by multichannel pipettes. After incubation at 37 °C for 1 h, the absorbance of the solution at 450 nm (sample) and 655 nm (reference) was measured using an Epoch microplate reader (BioTek Instruments, Winooski, VT, USA). The cell viabilities of HeLa cells after treatment various concentration of DET-PRX at pH 7.4 or pH 5.5 were examined according to the procedure as described above, but using DMEM adjusted its pH by the addition of HCl.

Stabilities of siRNA complexes in DMEM

siRNA complexes (siRNA 0.26 μg) in DMEM (20 μL) were incubated at 37 °C for 4h, and 6 × loading buffer (Takara Bio, Shiga, Japan) 4 μL was added to each siRNA solution. Gel electrophoresis was performed at room temperature in Tris-borate ethylenediaminetetraacetic acid (EDTA) buffer (TBE, 45 mM Tris-borate, 1 mM EDTA, pH 8.0 [0.5 × TBE]) on a 2% (w/v) agarose 21 gel at 100 V for 30 min. The gel was stained with ethidium bromide. The siRNA bands were visualized using an Amersham Typhoon scanner (FLA-9000, Fujifilm, Tokyo, Japan).

GFP mRNA silencing detected by RT-qPCR

Total RNA (500 ng) isolated using TRIzol reagent (Thermo Fisher Scientific K.K., Tokyo, Japan) from HeLa/GFP cells 48 h after treatment of siRNA, gapmer, or Cpf1 RNP complexes described in the previous section was reverse transcribed by using ReverTra Ace qPCR RT Kit (TOYOBO, Tokyo, Japan). qPCR was performed on a CFX/96/384/Connect (BIO RAD) using 2 μL of cDNA for each sample. The THUNDERBIRD SYBR qPCR mix (Takara Bio, Shiga, Japan) was used to detect products with 10 μM of the following primer sets. GFP: 5′-

CCGGTGGTGCAGATGAACTT-3′ and 5′-ATGGTGAGCAAGGGCGAG-3′. Human GAPDH: 5′-GCACCGTCAAGGCTGAGAAC-3′ and 5′-CTCCCAGGTGTCATCAGCAG-3′. qPCR was performed at 95 °C initially for 30 s followed by 40 cycles of 95 °C × 5 s, 55 °C × 30 s, and 72 °C × 30 s. The relative amount of cDNA in each sample was normalized to the expression of GAPDH, and the melt curve was used to verify specificity.

T7E1 assay

Genomic DNA of HeLa/GFP cells after treatment Cpf1 RNP complexes was extracted by using MightyPrep reagent for DNA (Takara Bio, Shiga, Japan). GFP genes were amplified by using MightyAmp DNA Polymerase Ver.3 (Takara Bio, Shiga, Japan) with a following primer set for GFP gene: 5′-ATGGTGAGCAAGGGCGAG-3′ and 5′-CCGGTGGTGCAGATGAACTT-3′. Obtained PCR products were purified by using NucleoSpin gel and PCR Clean-up (Takara Bio, Shiga, Japan), denatured, re-annealed, and digested by T7E1 by using the T7 endonuclease I assay kit (GeneCopoeia, Inc., Rockville, USA). Gel electrophoresis was performed as described in the previous section, but for 40 min using 4% agarose S21 gel. The integral value of each band was quantified by ImageJ software designed at National institute of Health (Bethesda, MD). Indels frequency (%) was calculated by relating the band strength of the specific cleavage product using the following relationship: Indels (%) = $100 \times (1 - (1 - \text{fraction cleaved})^{1/2})$ [2].

Measurement of physicochemical properties

In total, 200 μL of carbamate-Cys-DET-PRX complex solution was diluted with solvents (800 μL), such as HBSS (pH 7.4), acetate buffer (pH 5.5), or HBSS (GSH: 2 mM) in a 1.5 mL Eppendorf

tube. The diameters and ξ-potentials of the carbamate-Cys-DET-PRX complexes were measured using a Zetasizer Nano ZS apparatus (Malvern Instruments, Malvern, UK).

Drug releases from 5G particles in response to GSH

5G/siRNA (siRNA 0.26 μg), 5G/ASO (ASO 0.52 μg), 5G/mRNA (mRNA 0.25 μg), 5G/β-Gal (β-Gal 5 μg), or 5G/Cpf1 RNP (crRNA 0.4 μg) was prepared in HBSS (15 μL). Heparin sodium salt (Nacalai Tesque, Kyoto, Japan) alone solution or Heparin sodium salt and GSH solution was added to each sample and incubated at r.t., for 30 min. The final concentration of heparin sodium salt and GSH are set at 3.5 (w/w/) against loaded nucleic acids and/or protein and 2 mM, respectively. Gel electrophoresis was performed as described in the previous section, but for 40 min using 2% agarose S gel for mRNA and Cpf1 RNP and for 40 min using 1.8% agarose S gel for β-Gal. For staining β-Gal bands, Coomassie Brilliant Blue (CBB)-G250 (Tokyo Chemical Industry Co., Ltd., Tokyo, Japan) was used instead of ethidium bromide.

Data analysis

Data are presented as the mean ± standard error. A significance test was performed using the method proposed by Scheffe. $P < 0.05$ was considered statistically significant.

Supplementary Figures and Tables

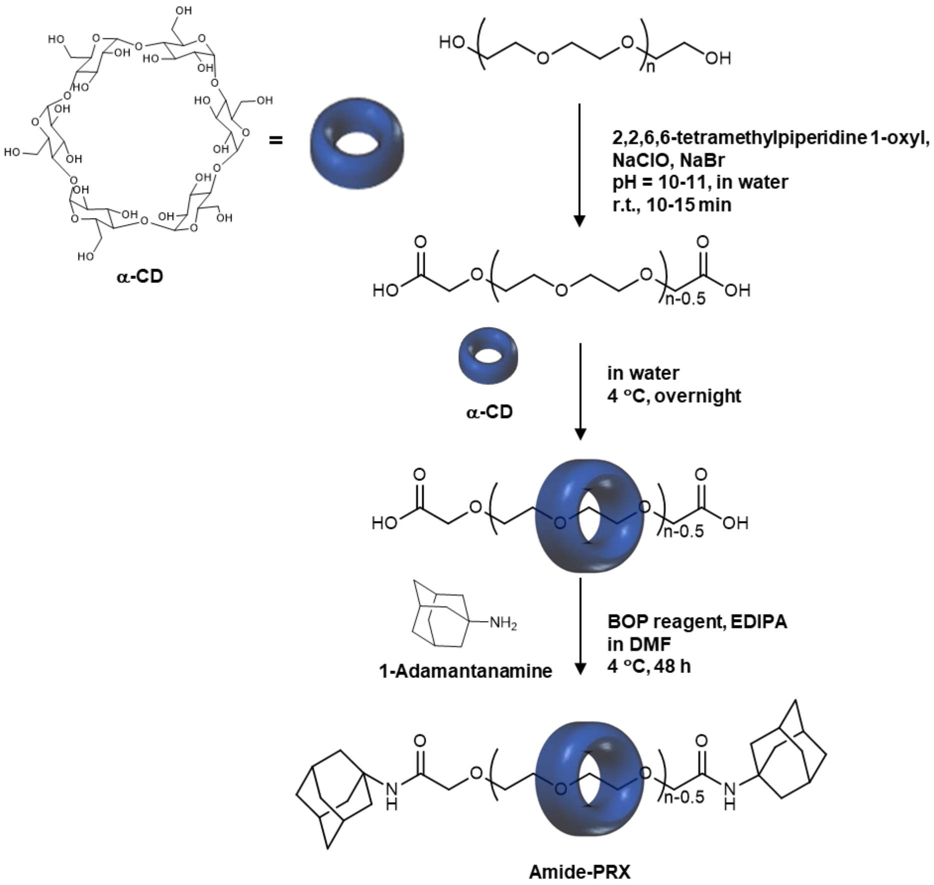

Figure S1. Pathway for the preparation of amide-PRX. BOP: benzotriazol-1-yloxytris(dimethylamino)phosphonium hexafluorophosphate reagent. EDIPA: *N*-ethyldiisopropylamine. DMF: dimethylformamide.

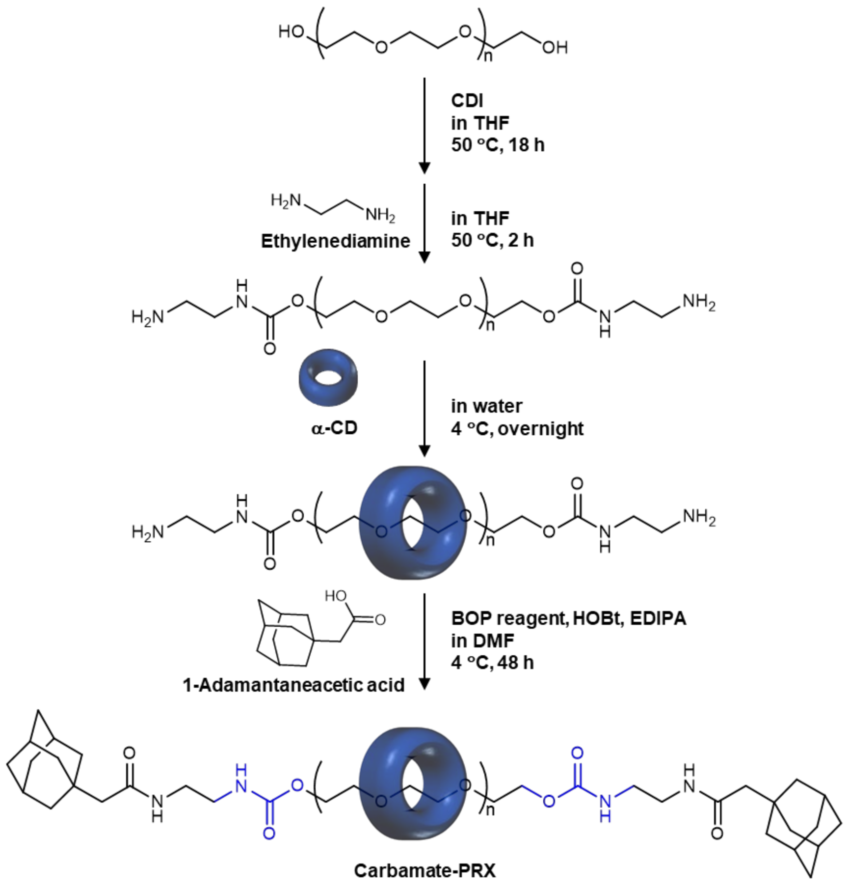

Figure S2. Pathway for the preparation of carbamate-PRX. CDI: *N*,*N*-carbonyldiimidazole. THF: tetrahydrofuran. HOBt: 1-hydroxybenzotriazole.

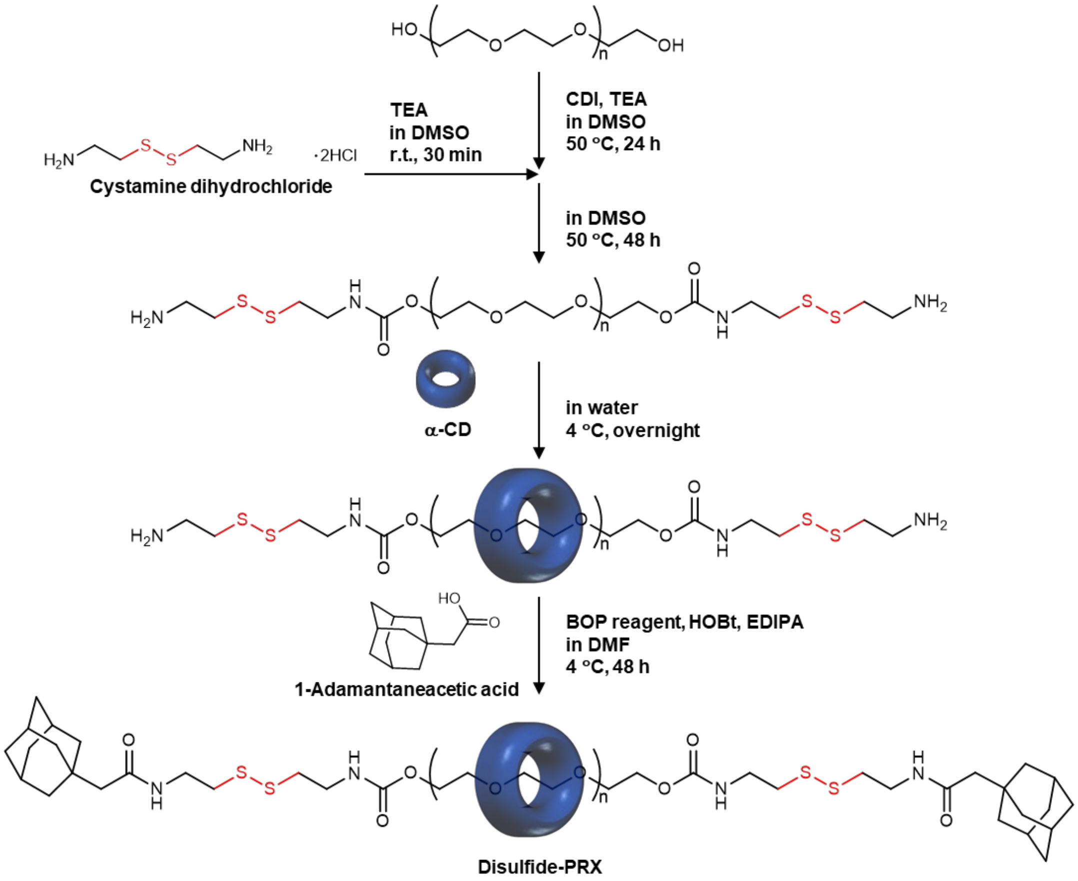

Figure S3. Pathway for the preparation of disulfide-PRX. TEA: triethylamine. DMSO: dimethyl sulfoxide.

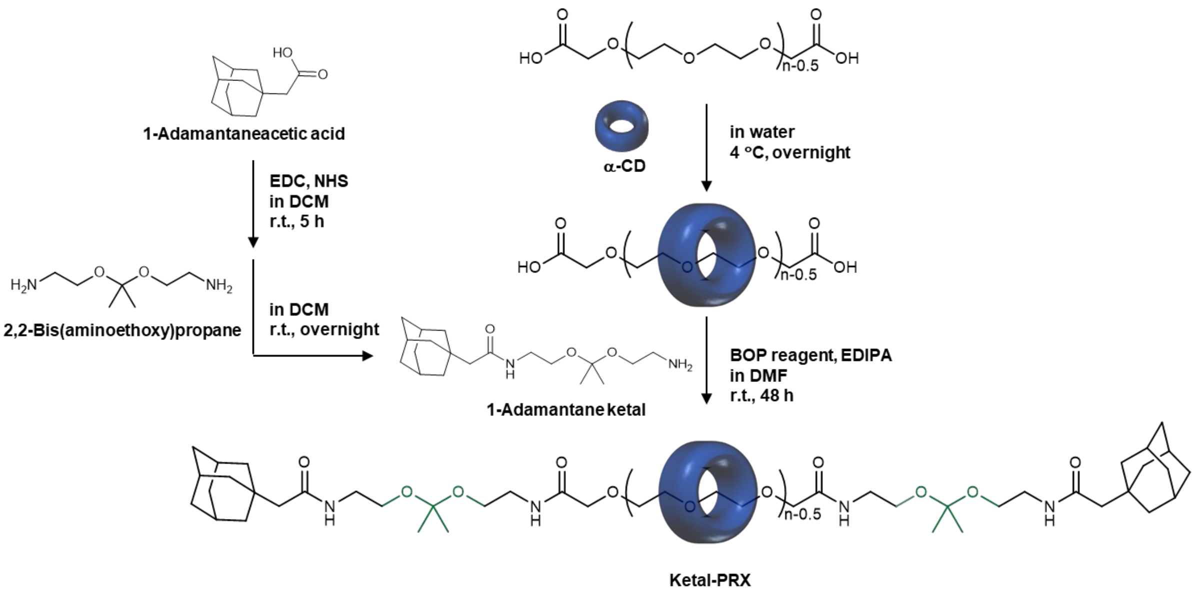

Figure S4. Pathway for the preparation of ketal-PRX. EDC: *N′*-ethylcarbodiimide hydrochloride. NHS: *N*-hydroxysuccinimide. DCM: dichloromethane.

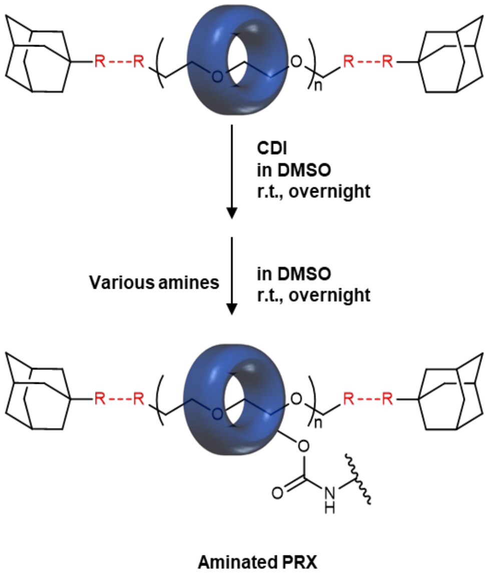

Figure S5. Pathway for the preparation of various aminated PRXs. The chemical structures of modified amino groups are shown in Figure 2.

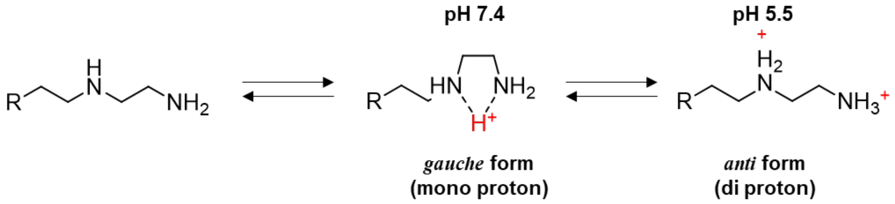

Figure S6. Transformation of the DET unit from the *gauche* form (mono proton) to the *anti* form (di proton) at acidic pH in late endosomes.

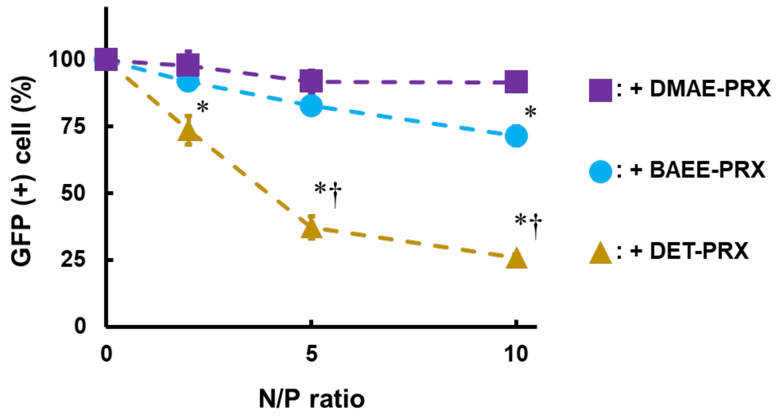

Figure S7. Effects of N/P ratio on the RNAi effects of aminated PRXs/siGFP in HeLa/GFP cells (n = 6). [siRNA] = 100 nM. *$p < 0.05$ *vs.* + DMAE-PRX. †$p < 0.05$ *vs.* + BAEE-PRX.

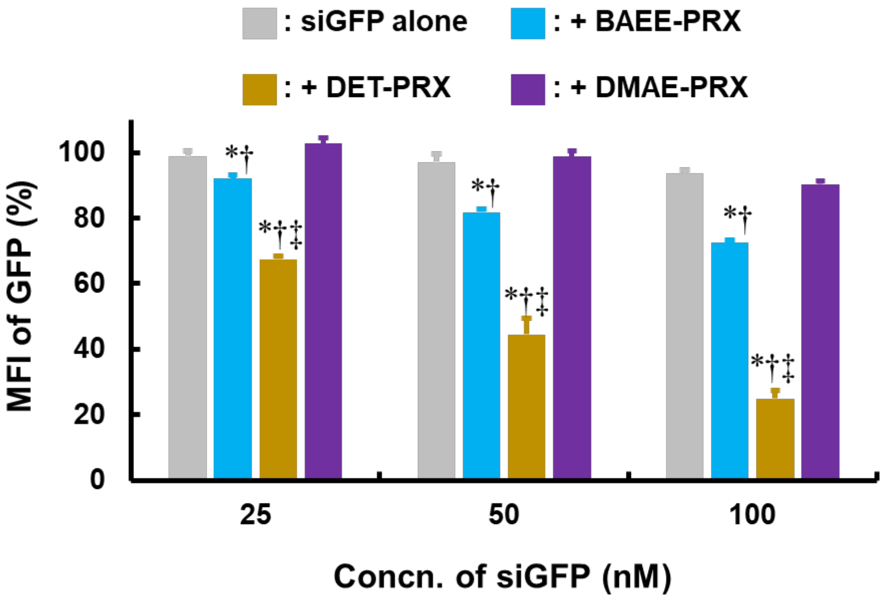

Figure S8. MFI of GFP in HeLa/GFP cells after treatment of various siGFP complexes ($n = 6$). *$p < 0.05$ *vs.* siRNA alone. †$p < 0.05$ *vs.* + DMAE-PRX. ‡ $p < 0.05$ *vs.* + BAEE-PRX.

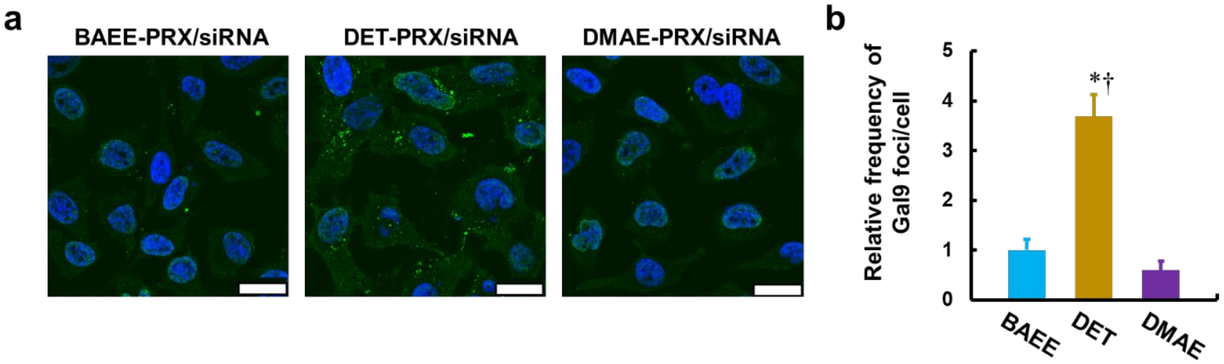

Figure S9. Gal9 recruitment in HeLa cells after treatment of BAEE-, DET-, or DMAE-PRX/siRNA. (a) Confocal image of Gal9 recruitment. Green: Gal9. Blue: nucleus (Hoechst 33342). Scale bar = 25 µm. (b) Relative frequency of Gal9 foci/cell (62–70 cells). The frequency of Gal9 foci/cell with BAEE-PRX/siRNA was set at 1.0. *p < 0.05 *vs.* BAEE-PRX/siRNA. †p < 0.05 *vs.* DMAE-PRX/siRNA.

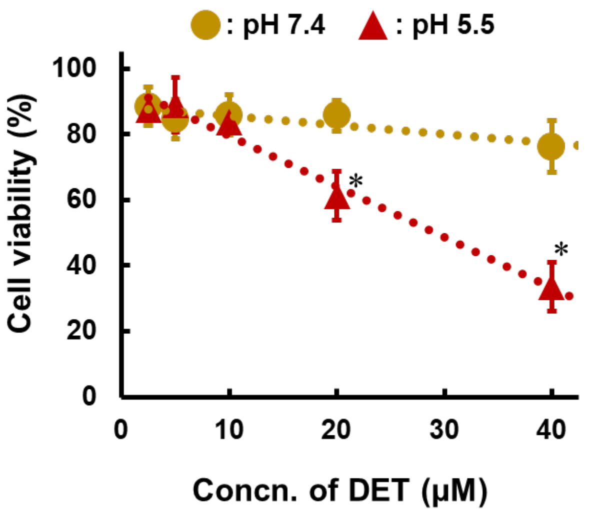

Figure S10. Cell viabilities of HeLa cells after treatment of DET-PRX at pH 7.4 or pH 5.5 (n = 3–4). *$p < 0.05$ *vs.* pH 7.4.

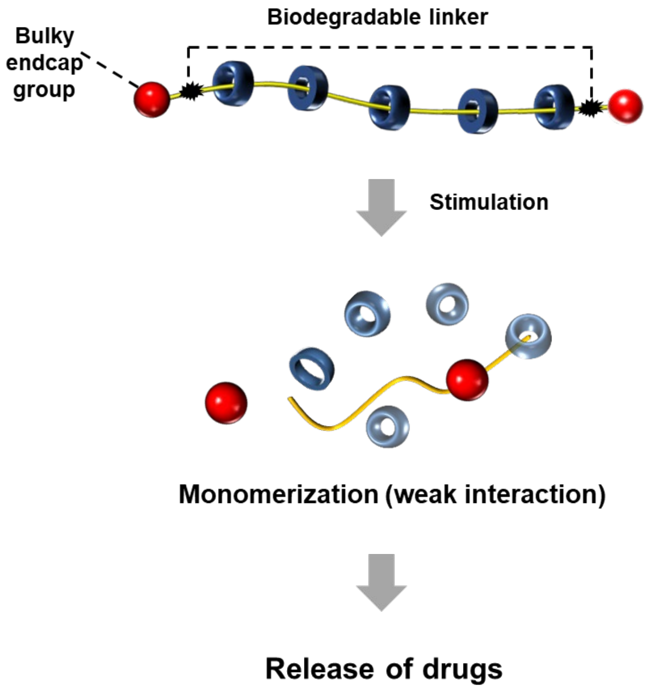

Figure S11. Scheme representing the endcap-degradable PRX and drug release.

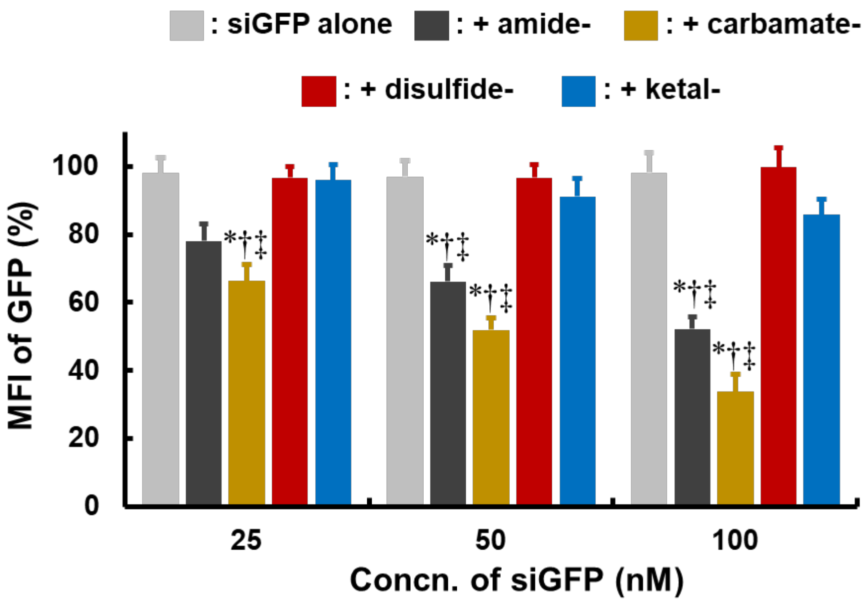

Figure S12. MFI of GFP in HeLa/GFP cells after treatment of various DET-PRX/siGFP ($n = 6$). *$p < 0.05$ *vs.* siRNA alone. †$p < 0.05$ *vs.* + disulfide-DET-PRX. ‡ $p < 0.05$ *vs.* + ketal-DET--PRX.

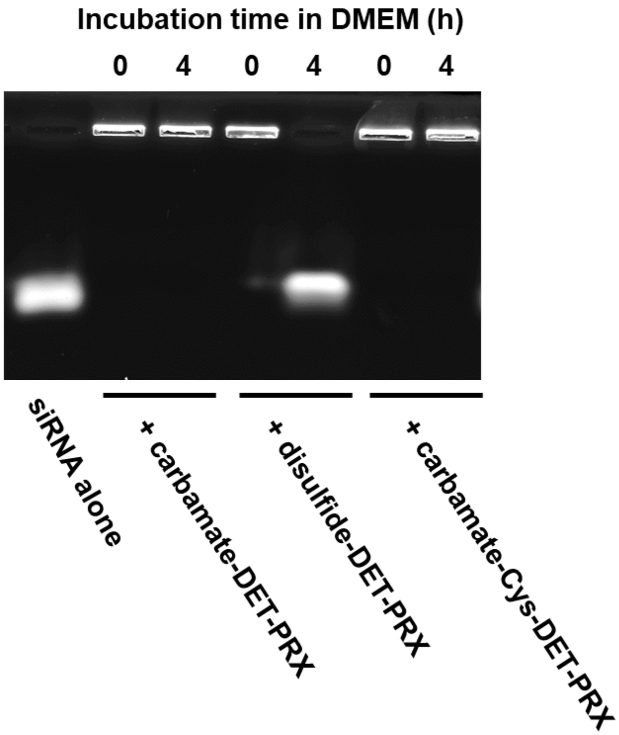

Figure S13. Stabilities of siRNA complexes after incubation in DMEM at 37 °C for 4 h.

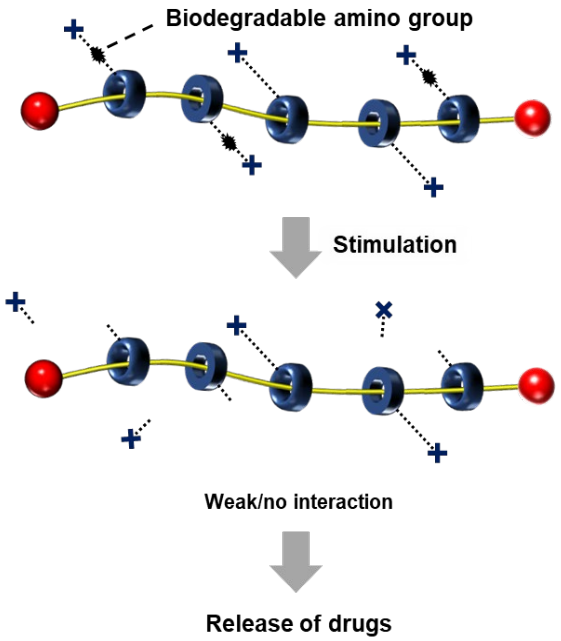

Figure S14. Scheme representing the amine-degradable PRX and drug release.

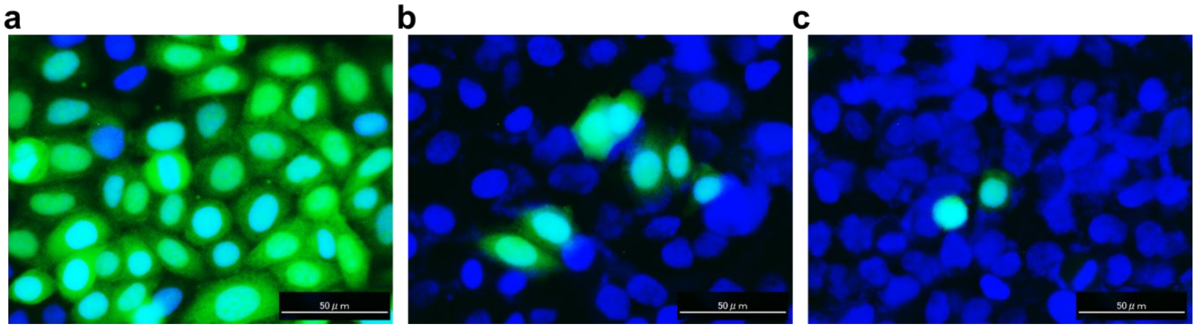

Figure S15. Fluorescence image of HeLa/GFP cells after treatment of siGFP complexes. [siGFP] = 100 nM. (a) control. (b) Lipo2000/siGFP. (c) carbamate-Cys-DET-PRX/siGFP. Green: GFP. Blue: nucleus (Hoechst 33342). Scale bar = 50 μm.

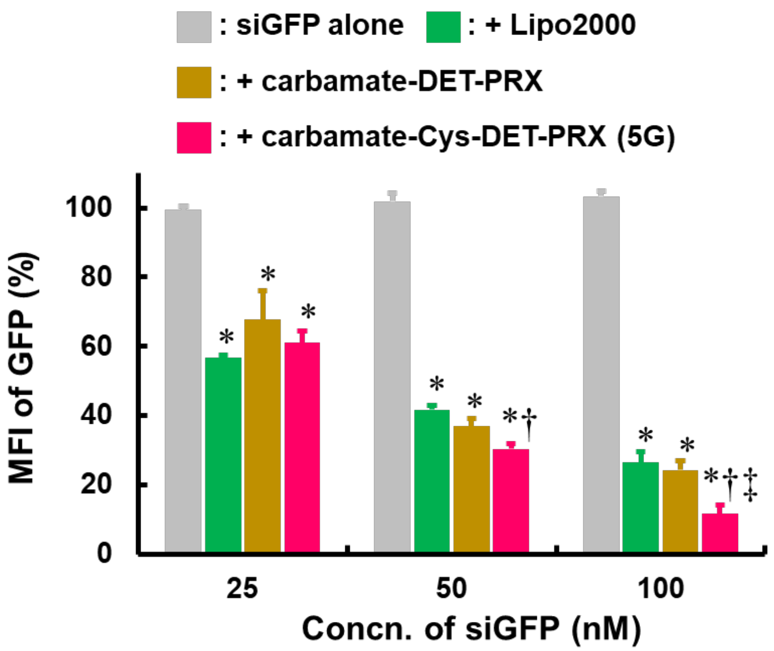

Figure S16. MFI of GFP in HeLa/GFP cells after treatment of Lipo2000/siGFP, carbamate-DET-PRX/siGFP, or carbamate-Cys-DET-PRX/siGFP (n = 6). *$p < 0.05$ *vs.* siRNA alone. †$p < 0.05$ *vs.* + Lipo2000. ‡ $p < 0.05$ *vs.* + carbamate-DET-PRX/siGFP.

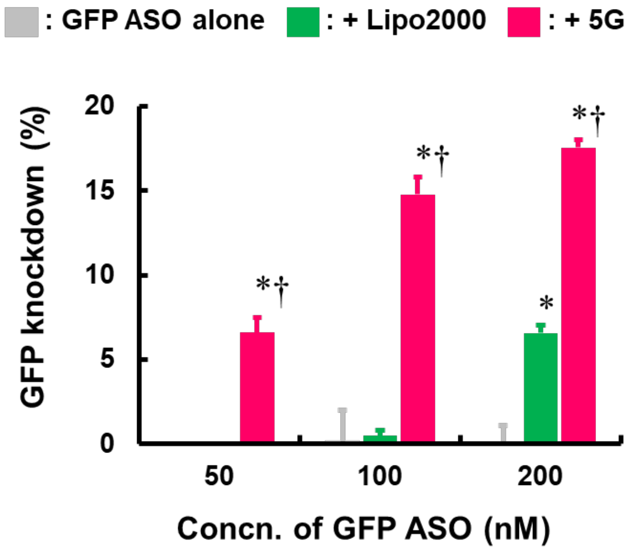

Figure S17. Effects of GFP knockdown on GFP ASO complexes with Lipo2000 or 5G in HeLa/GFP cells ($n = 6$). *$p < 0.05$ *vs*. GFP ASO. †$p < 0.05$ *vs*. + Lipo2000.

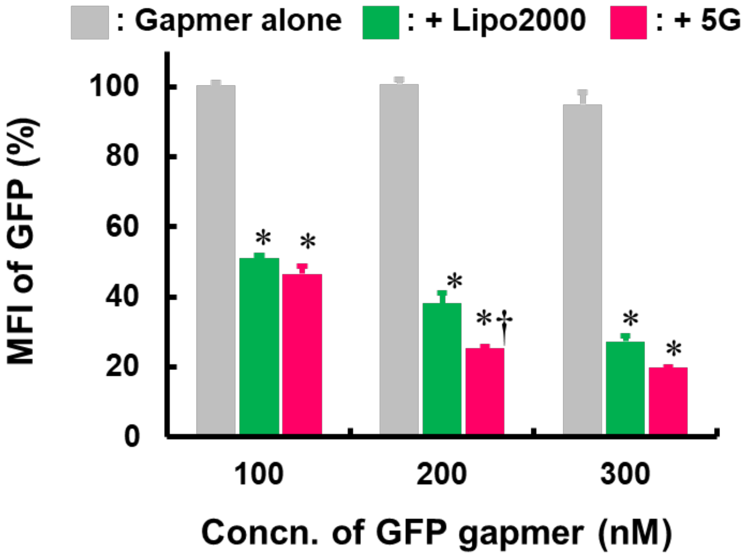

Figure S18. MFI of GFP in HeLa/GFP cells after treatment of various GFP gapmer complexes ($n = 6$). *$p < 0.05$ *vs*. GFP gapmer alone. †$p < 0.05$ *vs*. + Lipo2000.

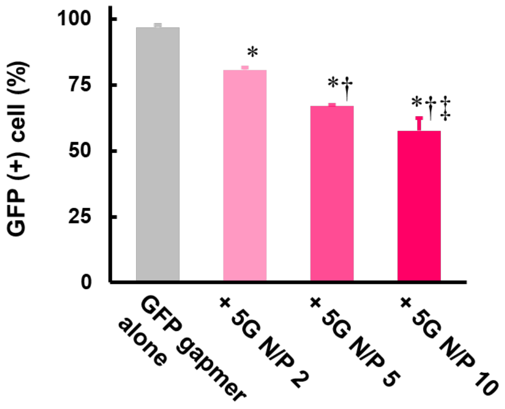

Figure S19. Effects of N/P ratio on the knockdown effects of 5G/GFP gapmer (*n* = 3). [Gapmer] = 100 nM. *$p < 0.05$ *vs*. GFP gapmer alone. †$p < 0.05$ *vs*. + 5G N/P 2. ‡$p < 0.05$ *vs*. + 5G N/P 5.

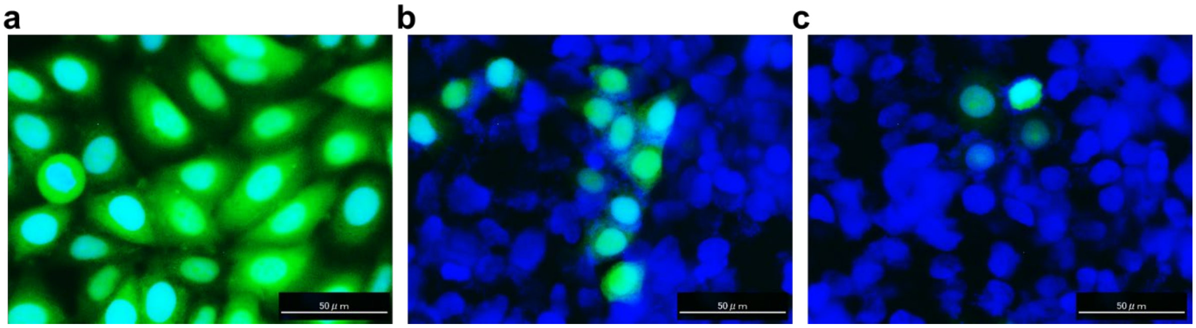

Figure S20. Fluorescence image of HeLa/GFP cells after treatment of GFP gapmer complexes. [GFP gapmer] = 300 nM. (a) control. (b) Lipo2000/GFP gapmer. (c) 5G/GFP gapmer. Green: GFP. Blue: nucleus (Hoechst 33342). Scale bar = 50 μm.

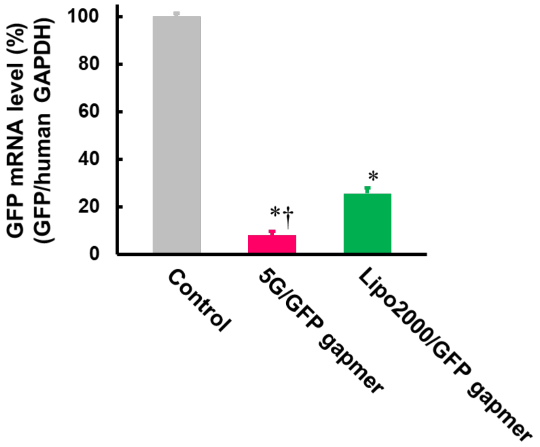

Figure S21. GFP mRNA silencing of GFP gapmer complexes in HeLa/GFP cells detected by RT-qPCR (n = 3). [GFP gapmer] = 300 nM. The relative expression level of GFP/human GAPDH mRNA of non-treated control was set at 100 %. *$p < 0.05$ *vs.* control. †$p < 0.05$ *vs.* Lipo2000/GFP gapmer.

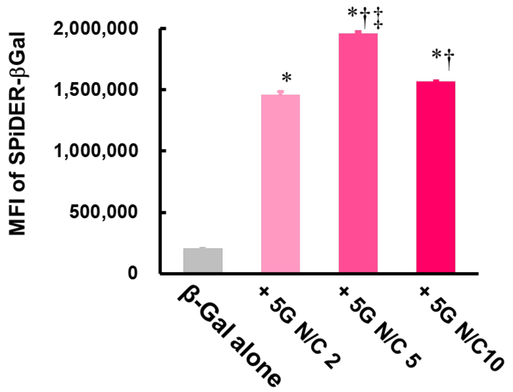

Figure S22. Effects of N/C ratio on the intracellular activity of 5G/β-Gal (n = 3). [β-Gal] = 25 nM. *$p < 0.05$ *vs.* β-Gal alone. †$p < 0.05$ *vs* + 5G N/C 2. ‡$p < 0.05$ *vs* + 5G N/C 10.

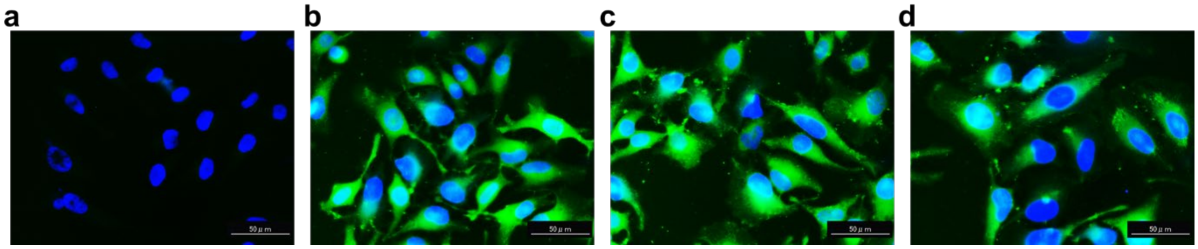

Figure S23. Fluorescence image of HeLa cells after treatment of 5G/β-Gal and SPiDER-βGal staining. [β-Gal] = 25 nM. (a) control. (b) 5G/β-Gal N/C 2. (c) 5G/β-Gal N/C 5. (d) 5G/β-Gal N/C 10.Green: hydrolyzed SPiDER-βGal. Blue: nucleus (Hoechst 33342). Scale bar = 50 μm.

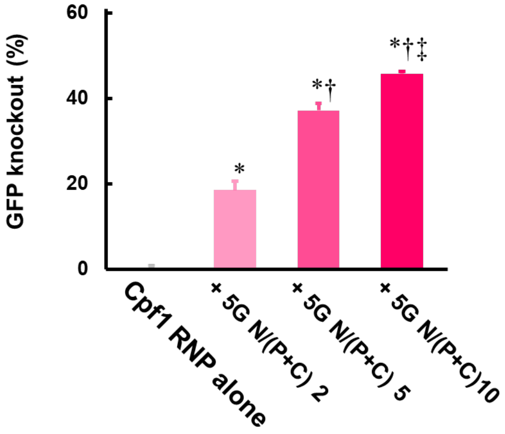

Figure S24. Effects of N/(P+C) ratio on the genome editing of 5G/Cpf1 RNP ($n = 3$). [Cpf1 RNP] = 29.2 nM. *$p < 0.05$ *vs.* Cpf1 RNP alone. †$p < 0.05$ *vs.* + 5G N/(P+C) 2. ‡$p < 0.05$ *vs.* + 5G N/(P+C) 5.

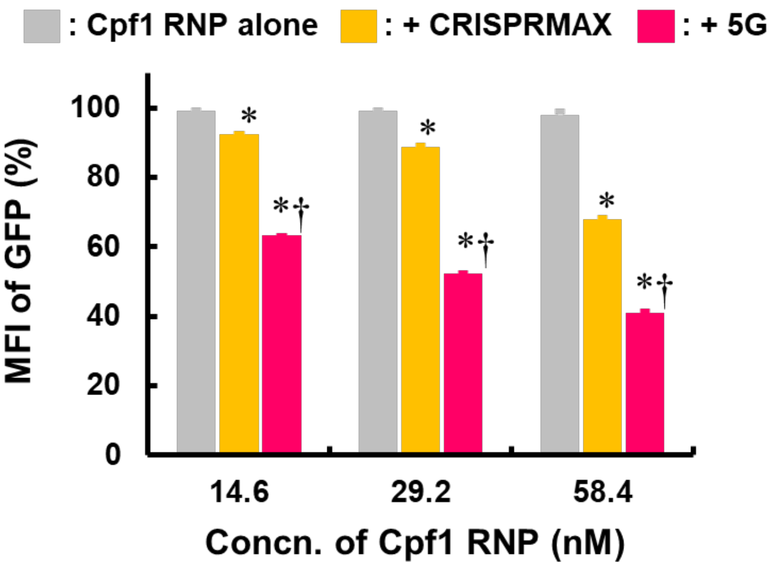

Figure S25. MFI of GFP in HeLa/GFP cells after treatment of various Cpf1 RNP complexes (n = 3). *$p < 0.05$ *vs.* Cpf1 RNP alone. †$p < 0.05$ *vs.* + CRISPRMAX.

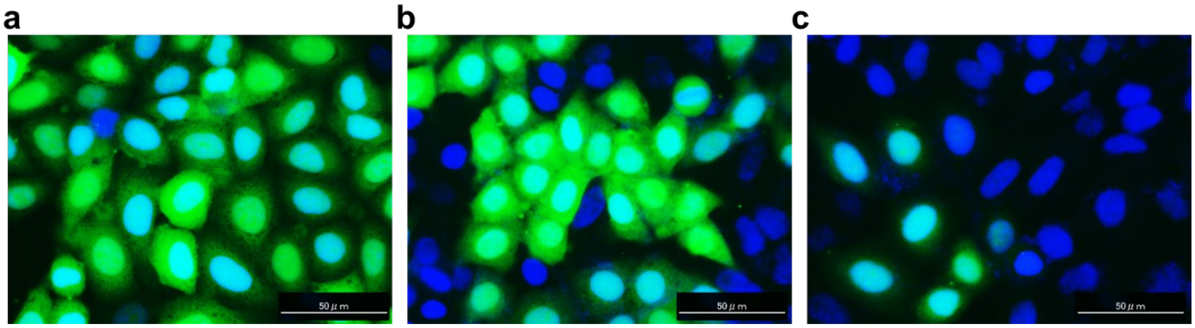

Figure S26. Fluorescence image of HeLa/GFP cells after treatment of Cpf1 RNP complexes. [Cpf1 RNP] = 58.4 nM. (a) control. (b) CRISPRMAX/Cpf1 RNP. (c) 5G/Cpf1 RNP: GFP. Blue: nucleus (Hoechst 33342). Scale bar = 50 μm.

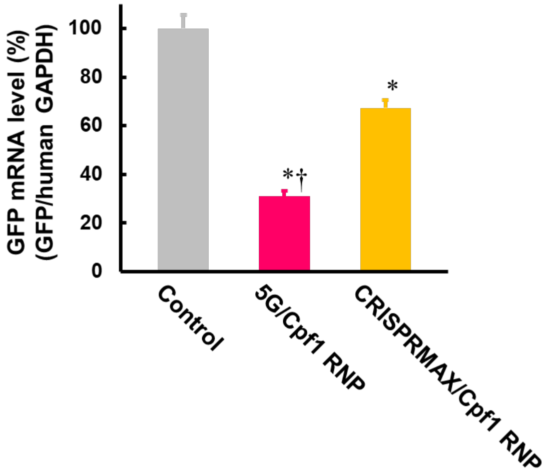

Figure S27. GFP mRNA silencing of Cpf1 RNP complexes in HeLa/GFP cells detected by RT-qPCR (n = 3). [Cpf1 RNP] = 58.4 nM. The relative expression level of GFP/human GAPDH mRNA of non-treated control was set at 100 %. *$p < 0.05$ *vs.* control. †$p < 0.05$ *vs.* CRISPRMAX/Cpf1 RNP.

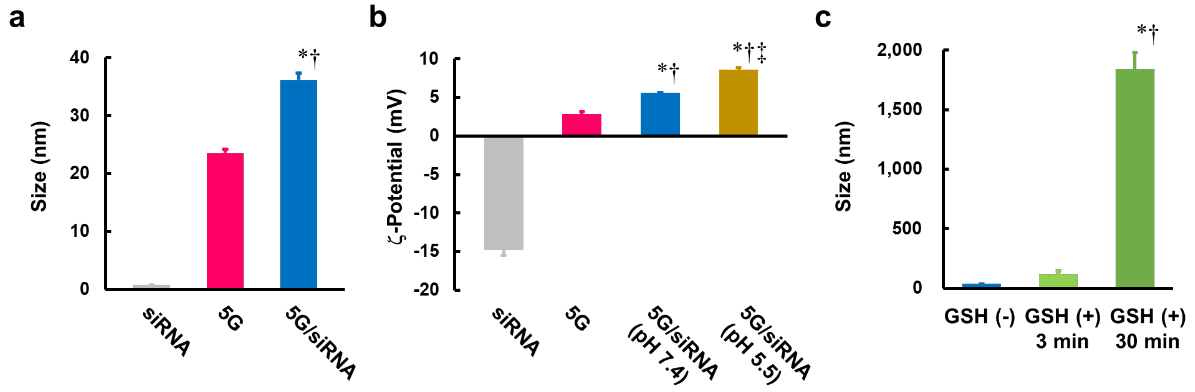

Figure S28. Multi-step transformable properties of 5G with siRNA. (a) Sizes (n = 6) and (b) ξ-potentials (n = 4–5) of siRNA, 5G, and 5G/siRNA in HBSS buffer (pH 7.4) or acetate buffer (pH 5.5). *$p < 0.05$ *vs.* siRNA. †$p < 0.05$ *vs* 5G. ‡$p < 0.05$ *vs.* 5G/siRNA (pH 7.4). (c) Effect of GSH on the size of 5G/siRNA (n = 4–6). [GSH] = 2 mM. *$p < 0.05$ *vs.* GSH (-). †$p < 0.05$ *vs.* GSH (+), 3 min.

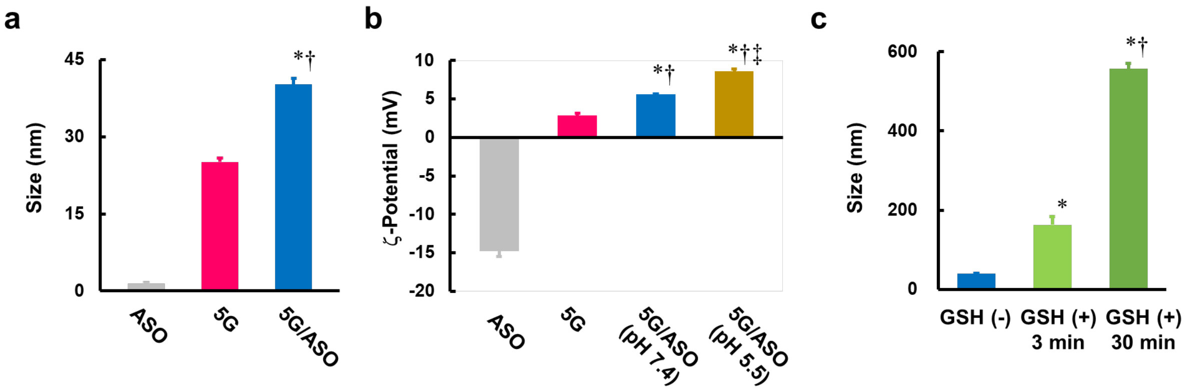

Figure S29. Multi-step transformable properties of 5G with ASO. (a) Sizes and (b) ξ-potentials of ASO, 5G, and 5G/ASO in HBSS buffer (pH 7.4) or acetate buffer (pH 5.5) (n = 5–6). *$p < 0.05$ *vs.* ASO. †$p < 0.05$ *vs* 5G. ‡$p < 0.05$ *vs.* 5G/ASO (pH 7.4). (c) Effect of GSH on the size of 5G/ASO. (n = 5–6) [GSH] = 2 mM. *$p < 0.05$ *vs.* GSH (-). †$p < 0.05$ *vs.* GSH (+), 3 min.

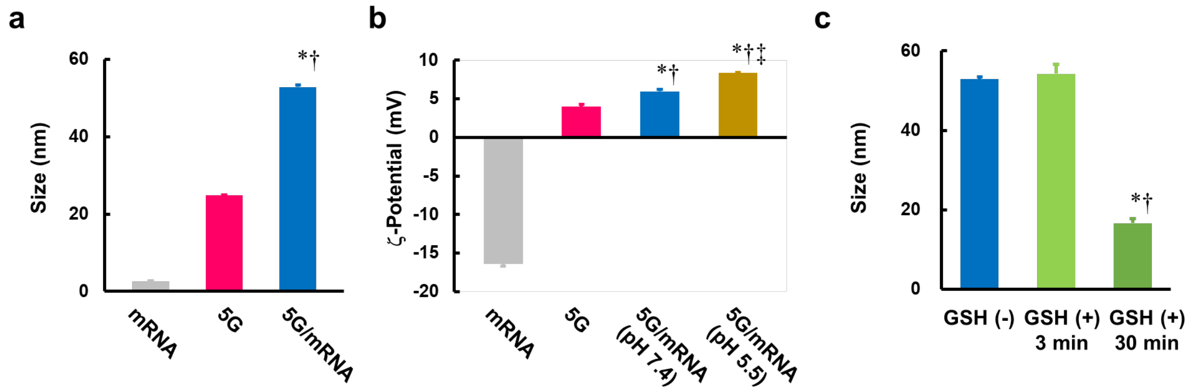

Figure S30. Multi-step transformable properties of 5G with mRNA. (a) Sizes and (b) ξ-potentials of mRNA, 5G, and 5G/mRNA in HBSS buffer (pH 7.4) or acetate buffer (pH 5.5) (n = 3–4). *$p < 0.05$ *vs.* mRNA. †$p < 0.05$ *vs.* 5G. ‡$p < 0.05$ *vs.* 5G/mRNA (pH7.4). (c) Effect of GSH on the size of 5G/mRNA (n = 3–4). [GSH] = 2 mM. *$p < 0.05$ *vs.* GSH (-). †$p < 0.05$ *vs.* GSH (+), 3 min.

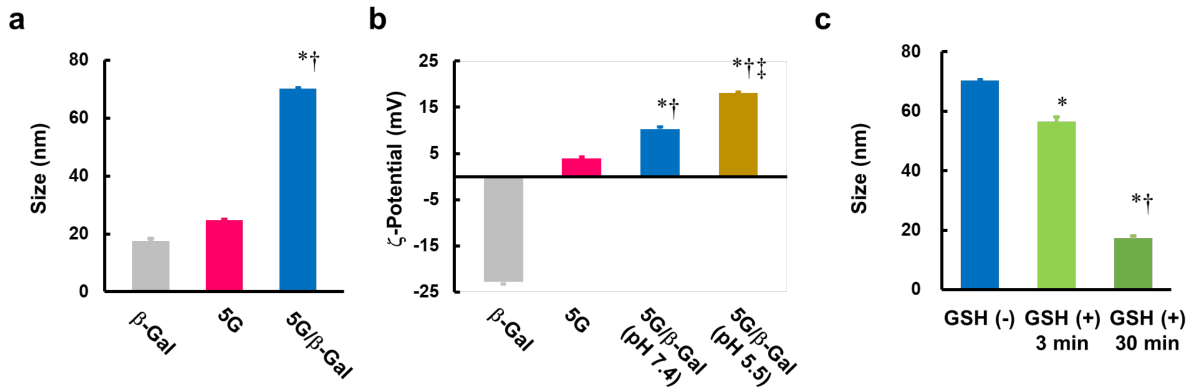

Figure S31. Multi-step transformable properties of 5G with β-Gal. (a) Sizes and (b) ξ-potentials of β-Gal, 5G, and 5G/β-Gal in HBSS buffer (pH 7.4) or acetate buffer (pH 5.5) (n = 3–4). *p < 0.05 *vs.* β-Gal. †p < 0.05 *vs.* 5G. ‡p < 0.05 *vs.* 5G/β-Gal (pH 7.4). (c) Effect of GSH on the size of 5G/β-Gal (n = 3). [GSH] = 2 mM. *p < 0.05 *vs.* GSH (-). †p < 0.05 *vs.* GSH (+), 3 min.

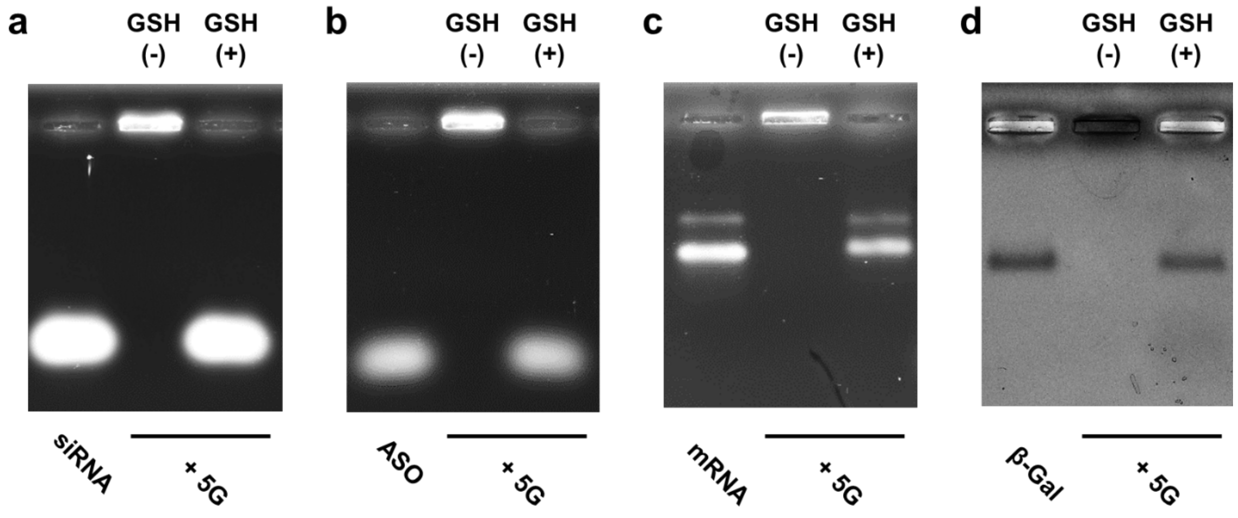

Figure S32. Agarose gel electrophoresis of (a) 5G/siRNA, (b) 5G/ASO, (c) 5G/mRNA, and (d) 5G/β-Gal with or without GSH (2 mM) treatment for 30 min.

Table S1. Sequences of nucleic acids used in this study.

Compound	Sequence
GFP targeted siRNA (siGFP)	sense: 5′-gcaagcugacccugaaguucauTT-3′<br>antisense: 5′-augaacuucagggucagcuugccg-3′
GFP targeted ASO (GFP ASO)	5′-TTGCCGGTGGTGCAGATAAA-3′
GFP targeted gapmer (GFP gapmer)	5′-**GAACTTCAGGGTCAGC**-3′
GFP targeted crRNA*	5′- CCTGGTCGAGCTGGACGGCGACG-3′

small letters: RNA bases; CAPITAL LETTERS: DNA bases; red font: locked nucleic acids (LNAs); **bold font**: phosphorothioate (PS) bonds.

*The targeted sequence of *GFP* gene is shown.

Table S2. Characterization of aminated PRXs used in this study [1]. [a)]

Compound	No. of α-CD	Coverage (%)	No. of amino units/CD	Molecular weight (kDa)
Carbamate-BAEE-PRX	61.6	27.1	3.4	111
Carbamate-DMAE-PRX	61.6	27.1	3.8	107
Carbamate-DET-PRX	61.6	27.1	3.3	107
Amide-DET-PRX	72.1	31.7	3.0	112
Disulfide-DET-PRX	52.5	23.1	3.6	95
Ketal-DET-PRX	60.2	26.5	3.3	99
Carbamate-Cys-DET-PRX	61.6	27.1	2.5 (Cys:DET = 1:1)	100

[a)] The data were characterized in ref [1].

Table S3. Application of aminated PRXs for the various biopharmaceutics.

Drug	Property	Carrier	Purpose	Efficacy	Safety
Cas9 RNP [1]	Cas9 protein (158 kDa) /sgRNA (100 mer)	5G	Intracellular delivery	++	+++
siRNA	RNA (19-23 bp)	5G	Intracellular delivery	+++	+++
ASO	ssDNA (13-20 mer)	5G	Intracellular delivery	+++	+++
mRNA	ssRNA (≥ 1000 mer)	5G	Intracellular delivery	+	+++
β-Gal	Protein (116 kDa, pI 5)	5G	Intracellular delivery	+++	+++
Cpf1 RNP	Cpf1 protein (156 kDa,) /crRNA (40-44 mer)	5G	Intracellular delivery	+++	+++
Insulin [3, 4]	Protein (5.8 kDa, pI 5)	(PEG)-BAEE-PRX	Stabilize & sustained release	+	+
Antibody (IgG) [3, 4]	Protein (150 kDa, pI ~ 7)	(PEG)-BAEE-PRX	Stabilize	+	+

Efficacy and safety +++: higher than commercially available positive control, ++: comparable to commercially available positive control, +: good but there was no comparison to commercially available positive controls.

Supplementary References

[1] T. Taharabaru, T. Kihara, R. Onodera, T. Kogo, K. Higashi, K. Moribe, T. Nakamura, K. Motoyama, T. Higashi, Polyrotaxane-based multi-step transformable materials for the delivery of Cas9 ribonucleoprotein, Appl. Mater. Today 27 (2022) 101488.

[2] D.Y. Guschin, A.J. Waite, G.E. Katibah, J.C. Miller, M.C. Holmes, E.J. Rebar, A rapid and general assay for monitoring endogenous gene modification, Methods Mol. Biol. 649 (2010) 247-56.

[3] T. Kogo, K. Utatsu, T. Taharabaru, R. Onodera, K. Motoyama, T. Higashi, Polyrotaxane-Based Supramolecular Material for Improvement of Pharmaceutical Properties of Protein Drugs, J. Pharm. Sci. (2022).

[4] K. Utatsu, T. Kogo, T. Taharabaru, R. Onodera, K. Motoyama, T. Higashi, Supramolecular polymer-based transformable material for reversible PEGylation of protein drugs, Mater. Today Bio. 12 (2021) 100160.
